# Lipoprotein-associated phospholipase A_2_: A paradigm for allosteric regulation by membranes

**DOI:** 10.1073/pnas.2102953118

**Published:** 2022-01-07

**Authors:** Varnavas D. Mouchlis, Daiki Hayashi, Alexis M. Vasquez, Jian Cao, J. Andrew McCammon, Edward A. Dennis

**Affiliations:** ^a^Department of Chemistry and Biochemistry, University of California San Diego, La Jolla, CA 92093-0601;; ^b^Department of Pharmacology, School of Medicine, University of California San Diego, La Jolla, CA 92093-0601

**Keywords:** phospholipase, lipoprotein, allosterism, membrane, lipid

## Abstract

Lipoprotein-associated phospholipase A_2_ (Lp-PLA_2_) associates with low- and high-density lipoproteins in human plasma and specifically hydrolyzes circulating oxidized phospholipids involved in oxidative stress. The association of this enzyme with the lipoprotein’s phospholipid monolayer to access its substrate is the most crucial first step in its catalytic cycle. The current study demonstrates unequivocally that a significant movement of a major helical peptide region occurs upon membrane binding, resulting in a large conformational change upon Lp-PLA_2_ binding to a phospholipid surface. This allosteric regulation of an enzyme’s activity by a large membrane-like interface inducing a conformational change in the catalytic site defines a unique dimension of allosterism. The mechanism by which this enzyme associates with phospholipid interfaces to select and extract a single phospholipid substrate molecule and carry out catalysis is key to understanding its physiological functioning. A lipidomics platform was employed to determine the precise substrate specificity of human recombinant Lp-PLA_2_ and mutants. This study uniquely elucidates the association mechanism of this enzyme with membranes and its resulting conformational change as well as the extraction and binding of specific oxidized and short acyl-chain phospholipid substrates. Deuterium exchange mass spectrometry coupled with molecular dynamics simulations was used to define the precise specificity of the subsite for the oxidized fatty acid at the *sn-2* position of the phospholipid backbone. Despite the existence of several crystal structures of this enzyme cocrystallized with inhibitors, little was understood about Lp-PLA_2_‘s specificity toward oxidized phospholipids.

## Significance

Lp-PLA_2_ is a physiologically important human enzyme and an inflammatory biomarker for assessing risk factors associated with cardiovascular diseases. It is associated with low- and high-density lipoproteins in human plasma and acts on the outside of the phospholipid monolayer that coats these particles, in stark contrast to traditional PLA_2_ enzymes that act on bilayer membranes. This study addresses the allosteric activation of Lp-PLA_2_ by phospholipid monolayers and membranes, its precise selectivity and specificity for particular oxidized and short acyl-chain phospholipid substrates not previously possible. Of particular importance, this work identifies and confirms by site-directed mutagenesis a phospholipid head-group binding pocket distinct from known drug inhibitor binding pockets that informs us about Lp-PLA_2_’s mechanism of action and creates opportunities for additional therapeutic approaches.

Peripheral proteins are membrane-associated proteins that reversibly bind to the surface of biological lipid bilayers. Despite the vital role of membrane-associated proteins in a variety of cellular processes, little is known about the membrane-association mechanism. Phospholipases A_2_ (PLA_2_) are membrane-associated enzymes for which interfacial binding plays a significant role in their biological activity and function. There are now six main types of PLA_2_, and each has a distinctive three-dimensional structure that contains a unique active site where the specific phospholipid substrate binds and an interfacial surface through which association with the membrane occurs (1, 2). Among them, human group VIIA lipoprotein-associated phospholipase A_2_ (GVIIA Lp-PLA_2_), also known as platelet-activating factor (PAF) acetylhydrolase (PAF-AH), constitutes an ideal system for studying protein–lipid and protein–inhibitor binding and interactions using hydrogen/deuterium exchange mass spectrometry (HD-XMS) and molecular dynamics (MD) simulations (3).

Lp-PLA_2_ is secreted in multiple inflammatory cells including macrophages exhibiting both anti-inflammatory and proinflammatory roles (4, 5). As its name indicates, Lp-PLA_2_ is associated with lipoproteins in human plasma for about 70% with low-density lipoprotein (LDL) and 30% with high-density lipoprotein (HDL) (6). This enzyme was found to hydrolyze the acetyl group at the *sn*-2 position of PAF, explaining its original name as PAF acetylhydrolase (7). It was also shown that Lp-PLA_2_ exhibits catalytic activity toward phosphatidylcholines (PCs) containing short acyl-chains, oxidized acyl-chains, and F2-isoprostanes esterified at the *sn*-2 position (8–10). The unique catalytic action of Lp-PLA_2_ makes this enzyme an attractive pharmacological target for atherosclerosis and cardiovascular diseases (11–14). Several crystal structures of Lp-PLA_2_ were reported either with or without inhibitors cocrystalized in the active site (15–18). Earlier HD-XMS studies revealed the peptide regions that mediate interactions with the membrane, HDL, and ApoA-I (19, 20). The present study constitutes a unique effort to elucidate the membrane-association mechanism, extraction and binding of several phospholipid substrates, and interactions of a potent inhibitor.

## Results

### Lp-PLA_2_ Structure and Active Site Properties.

The crystal structure of Lp-PLA_2_ revealed an α/β hydrolase fold with the catalytic Ser273 within a lipase consensus sequence of GXSXG (*SI Appendix*, Fig. S1) (15). The PLA_2_ active site was evolved to bind phospholipids, which are amphipathic molecules. As a result, a PLA_2_ active site consists of a hydrophilic region where the phospholipid headgroup binds and a hydrophobic region where the two phospholipid acyl-chains bind. We have shown for cytosolic, calcium-independent, and secreted (c, i, and s)PLA_2_ enzymes that there are two distinct hydrophobic subsites, one for each acyl chain of the substrate (21). However, each PLA_2_ active site has unique structural characteristics because each enzyme exhibits specificity for distinct phospholipid molecules (21). To define the Lp-PLA_2_ active site and understand its properties, we have employed SiteMap (22). The pocket with the highest SiteScore (1.05) has a volume of 325.5 Å^3^ and total solvent-accessible surface of 1,562.4 Å^2^. *SI Appendix*, Fig. S2*A* depicts the residues that are located within 5 Å of this pocket including Trp97, Leu107, Phe110, Leu121, Phe125, His151, Leu153, Phe156, Tyr160, Gln211, Glu214, Arg218, His272, Ser273, Phe274, Asp296, Trp298, His351, Gln352, Phe357, Ile365, and Leu369. The active site of Lp-PLA_2_ consists of a hydrophilic and a hydrophobic region. The hydrophilic region contains a classic catalytic triad of Ser273, His351, and Asp296, an oxyanion hole of Leu153 and Phe274 (backbone), and other residues including His151, Tyr160, Gln211, Glu214, Arg218, His272, Trp298, and Gln352, which are hydrogen-bond donors or acceptors (*SI Appendix*, Fig. S2*A*). The hydrophobic region includes residues such as Trp97, Leu107, Phe110, Leu121, Phe125, Phe156, Phe357, Ile365, and Leu369, which can form pi–pi stacking or van der Waals interactions. Several crystal structures of Lp-PLA_2_ were aligned to identify possible side chain conformational changes of the active site residues (*SI Appendix*, Fig. S2*B*) (15–18). However, no significant side chain conformational changes occurred, and thus the crystal structure with Protein Data Bank (PDB) ID 3D59 was used with resolution 1.5 Å. Mutations in several of these active site residues resulted in decreased in vitro activity, suggesting that these residues play an important role in binding and/or catalysis (*SI Appendix*, Fig. S2*C*).

### Binding and Interactions of SB-402564.

Potent and selective Lp-PLA_2_ inhibitors are useful tools for understanding the biological function of this enzyme with potential for developing new therapeutic agents for cardiovascular diseases (1). GlaxoSmithKline has developed Darapladib, which is a very potent pyrimidone Lp-PLA_2_ inhibitor with an inhibitory activity (IC_50_) of 0.25 nM (*SI Appendix*, Fig. S3) (23). Darapladib contains two substitutions at the pyrimidone ring that contain aromatic rings. SB-402564 is also a potent pyrimidone Lp-PLA_2_ inhibitor with an inhibitor activity (IC_50_) of 0.2 nM. Compared to Darapladib, SB-402564 contains an extra methoxy-dihydropyrimidine substitution at the pyrimidone ring (*SI Appendix*, Fig. S3). Understanding the binding and interactions of existing inhibitors is fundamental in developing new compounds with improved inhibitory properties. HD-XMS studies allowed us to capture the exchange rates of the backbone amide hydrogen atoms of Lp-PLA_2_ in the presence of SB-402564 and deuterium oxide. Three peptide regions including residues 105 to 110 (pink), 148 to 155 (green), and 346 to 356 (red) showed changes in the deuteration level upon binding of the inhibitor (*SI Appendix*, Fig. S4). Active site residues including Leu107, Phe110, Leu153, His351, and Gln352 are part of the peptide regions that were identified by HD-XMS to interact with the inhibitor, indicating that this pocket is likely the binding pocket for SB402564. In an effort to predict the binding mode of this inhibitor, rigid-receptor molecular docking was initially employed. This method does not allow any side chain flexibility for the residues of the active site, but it allows full flexibility for the inhibitor. Three binding modes were predicted that have quite different orientations in the Lp-PLA_2_ active site (*SI Appendix*, Fig. S5 *A*–*C*). In the first binding mode (BM1), the methoxy-dihydropyrimidine and the biphenyl chains of the inhibitor were placed in the hydrophobic region of the active site, while the fluorobenzyl chain was placed in the hydrophilic region (*SI Appendix*, Fig. S5*A*). The second binding mode (BM2) showed the fluorobenzyl chain in the hydrophobic region and the other two chains in the hydrophilic region (*SI Appendix*, Fig. S5*B*). Finally in the third binding mode (BM3), the biphenyl chain was placed in the hydrophobic region and the other two chains in the hydrophilic region (*SI Appendix*, Fig. S5*C*). Since it was not clear which was the correct binding mode of SB-402564, induced fit-receptor docking was also employed to further explore possible binding modes of the inhibitor. Induced fit-receptor docking allows side chain flexibility for the receptor and full flexibility for the inhibitor. The binding modes produced by induced fit-receptor docking were similar to the ones produced by rigid-receptor docking (*SI Appendix*, Fig. S5 *D*–*E*).

**Fig. 1.**
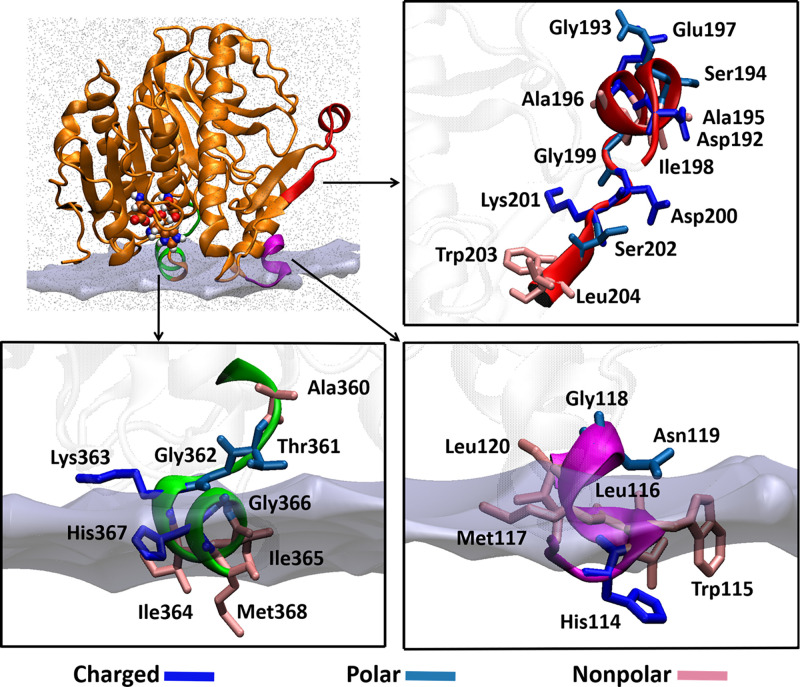
Peptide regions of Lp-PLA_2_ that interact with phospholipid vesicles and HDL. Residues 114 to 120 and 360 to 368 mediate Lp-PLA_2_ association with the membrane, while residues 192 to 204 were found to be involved in the association with HDL. These regions were used to generate an in silico enzyme–membrane model.

One of the main limitations of currently available molecular docking algorithms is that they do not consider the dynamic nature of the receptor. Conformational dynamics of a receptor plays a significant role in the binding and interactions of a ligand. To incorporate flexibility for the receptor, the relaxed complex scheme (RCS) was employed, which combines MD simulations with molecular docking (24, 25). Lp-PLA_2_ is a membrane-associated enzyme, and its structure contains an interfacial surface which mediates association with the membrane (3). According to previously published HD-XMS data (19, 20), peptide regions 114 to 120 and 360 to 368 had decreased exchange rates in the presence of phospholipid vesicles (Fig. 1). These residues are part of two amphipathic helices consisting of hydrophobic and hydrophilic residues. Amphipathic helices play a significant role in protein–membrane binding because their special properties enable them to overcome the water–lipid interfacial energy barrier (26). Several phospholipases including cPLA_2_, iPLA_2_, and sPLA_2_ were found to have similar helices as part of their interfacial surface (2, 21, 27). The third region showed decreased exchange rates in the presence of HDL, suggesting that this is the region through which Lp-PLA_2_ associates with lipoproteins. An in silico enzyme–membrane model was generated based on the HD-XMS data, which was solvated and subjected to 1-µs simulation (Fig. 1). Clustering analysis gave five main conformations that were used in docking calculations to predict the binding mode of SB-402564 in the Lp-PLA_2_ active site (*SI Appendix*, Fig. S6*A*). Three binding modes were predicted in which the methoxy-dihydropyrimidine chain of SB-402564 was placed in the hydrophilic area of the active site, while the other two chains were placed in the hydrophobic area (BM4-BM6, *SI Appendix*, Fig. S6*B*). In these three binding modes, the orientation of SB-402564 in the active site of Lp-PLA_2_ was different compared to the initial binding modes predicted by rigid and induced fit-receptor docking (BM1-BM3, *SI Appendix*, Fig. S5).

**Fig. 2.**
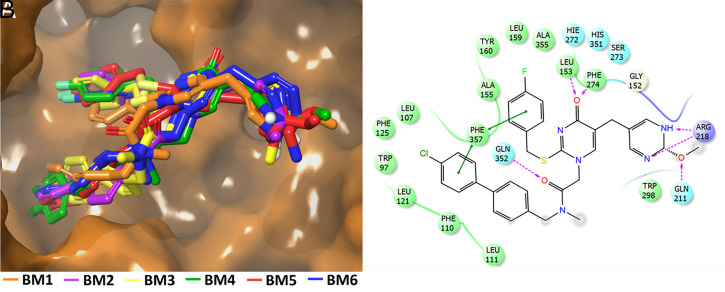
Optimized binding mode of SB-402564 after the MD simulations. (*A*) Each of the six docking binding modes adopted the same conformation and orientation in the Lp-PLA_2_ active site (Movies S1–S6); see the composite of all six (Movie S7). (*B*) Noncovalent interactions of the inhibitor with the active site residues are also shown.

Since we could not identify a predominant binding mode for SB-402564 using molecular docking and RCS, MD simulations were performed on each of the six binding modes in the presence of a membrane patch (Movies S1–S6). MD simulations provide full flexibility for the enzyme and the ligand, allowing them to adopt thousands of conformations and improve their binding and interactions. At the end of each simulation, SB-402564 adopted the same binding mode in terms of conformation, orientation, and interactions with the Lp-PLA_2_ active site (Fig. 2*A* and Movie S7). During the MD simulations, hydrogen-bonding interactions occurred between the oxygen atom of the methoxy-dihydropyrimidine ring with Gln211 and between the nitrogen atoms and Arg218 (Fig. 2*B*). The oxyanion hole (Leu153/Phe274) showed hydrogen bonding with the carbonyl oxygen atom of the pyrimidone ring. Hydrogen bonding also occurred between the amide oxygen atom on the biphenyl chain and Gln352. The biphenyl and fluorobenzyl chains were placed in the hydrophobic region of the active site participating in pi–pi aromatic interactions with residues such as Trp97, Phe110, Phe125, Tyr160, Phe357, Phe274, and Phe357. Aromatic–aliphatic interactions of the phenyl rings with residues Leu107, Leu111, Leu121, Ala155, Leu159, and Ala355 were also observed. The crystal structure of Lp-PLA_2_ cocrystallized with Darapladib was published soon after the MD simulations on SB-402564 were completed (18). It is worth noting that the biphenyl and fluorobenzyl chains of Darapladib in the crystal structure were oriented similarly to the ones of SB-402564 after the MD simulations (*SI Appendix*, Fig. S7). The optimized enzyme–inhibitor complexes produced by the MD simulations were utilized to redock SB-402564 in the active site of Lp-PLA_2_ to examine if rigid-receptor docking was able to reproduce the suggested by the MD simulations binding mode. The redocked binding modes were identical to the ones produced by the MD simulations (*SI Appendix*, Fig. S8). *SI Appendix*, Table S1 summarizes the theoretical binding score of rigid-receptor docking before and after the MD simulations.

### Substrate Specificity.

**Fig. 3.**
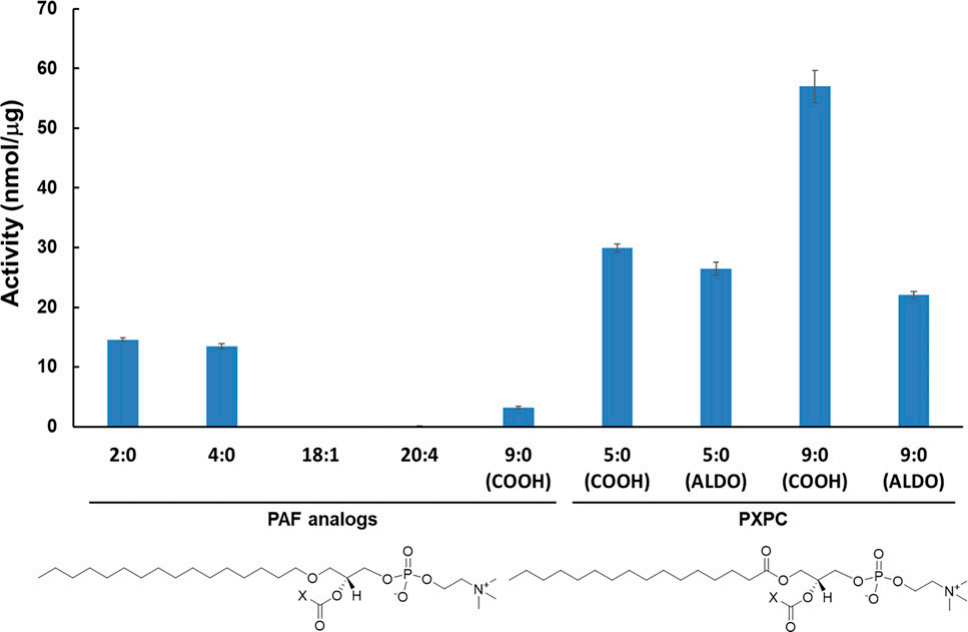
Enzymatic activity of Lp-PLA_2_ toward 100 µM of various PAF analogs and oxidized phospholipids.

A lipidomics assay using high-performance liquid chromatography, hydrophilic interaction chromatography, and multiple reaction monitoring was developed and successfully employed by our laboratory to study substrate specificity for c, i, and sPLA_2_ enzymes (21). This assay was further developed to perform dose–response inhibition studies on PLA_2_ inhibitors using a variety of phospholipid substrates and membrane-like mixtures of phospholipid species (28, 29). This assay has now been employed to perform substrate specificity studies on Lp-PLA_2_ on a variety of PAF analogs and oxidized phospholipids not feasible with traditional radioactive assays due to the lack of availability of radiolabeled substrates. Five PAF analogs and four oxidized phospholipids were tested in vitro as substrates for Lp-PLA_2_ (Fig. 3). Among the PAF analogs, 1-O-hexadecyl-2-acetyl-sn-glycero-3-phosphocholine (*sn*-2 acyl-chain: 2:0), also called PAF, was the optimal substrate for Lp-PLA_2_, followed by 1-O-hexadecyl-2-butyryl-sn-glycero-3-phosphocholine (*sn*-2 acyl-chain: 4:0) and 1-hexadecyl-2-azelaoyl-sn-glycero-3-phosphocholine [*sn*-2 acyl-chain: 9:0 (COOH)]. Lp-PLA_2_ showed minimal enzymatic activity toward 1-O-hexadecyl-2-oleoyl-sn-glycero-3-phosphocholine (*sn*-2 acyl-chain: 18:1) and 1-O-hexadecyl-2-arachidonoyl-sn-glycero-3-phosphocholine (*sn*-2 acyl-chain: 20:4). Lp-PLA_2_ exhibited higher activity toward oxidized phospholipids compared to PAF analogs. Among the oxidized phospholipids, 1-palmitoyl-2-azelaoyl-sn-glycero-3-phosphocholine [*sn*-2 acyl-chain: 9:0 (COOH)] was the optimal substrate for Lp-PLA_2_, followed by 1-palmitoyl-2-succinoyl-sn-glycero-3-phosphocholine [*sn*-2 acyl-chain: 5:0 (COOH)], 1-palmitoyl-2-(5′-oxo-valeroyl)-sn-glycero-3-phosphocholine [*sn*-2 acyl-chain: 5:0 (ALDO)], and 1-palmitoyl-2-(9′-oxo-nonanoyl)-sn-glycero-3-phosphocholine [*sn*-2 acyl-chain: 9:0 (ALDO)].

### Extraction and Binding of Substrates.

According to our in vitro results and consistent with the literature, Lp-PLA_2_ was found to act at the *sn*-2 position of PAF (7), short acyl-chain PCs (8), oxidized PCs (9), and phospholipids containing F2-isoprostanes esterified at the *sn*-2 position (10). The mechanism by which Lp-PLA_2_ extracts and binds a phospholipid molecule is still unknown since there are no available crystal structures of the enzyme cocrystallized with substrates. MD simulations guided by HD-XMS data have been successfully employed by our group to study binding and interactions of other human PLA_2_ enzymes with membranes and substrates (2, 21). Based on the experimental results, Lp-PLA_2_, showed significant enzymatic activity toward three PAF analogs and four oxidized PCs (Fig. 3). Seven MD simulations were performed on Lp-PLA_2_ in the presence of a membrane, and each simulation contained one of these substrates to identify the structural features that contribute to substrate specificity.

**Fig. 4.**
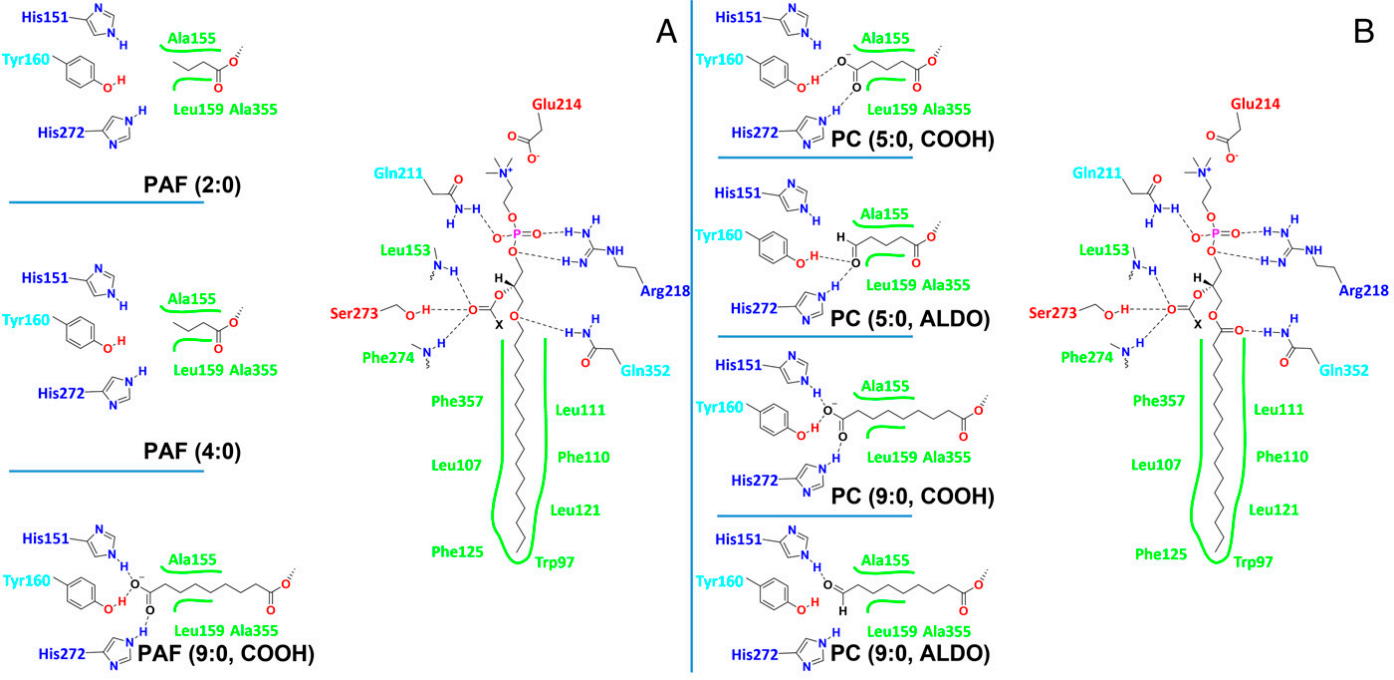
Binding interactions with the active site residues of Lp-PLA_2_ revealed by MD simulations for (*A*) PAF analogs (e.g., PAF-azelaoyl) and (*B*) oxidized PCs (e.g., azelaoyl-PC and F2-isoprostane-PC).

The MD simulations showed that the *sn*-1 chain binds to the lipophilic area of the active site interacting with residues Leu111, Phe110, Leu121, Trp97, Phe125, Leu107, and Phe357 (Fig. 4 *A* and *B*). The binding of this chain is similar in both PAF analogs and oxidized PCs. All the substrates contain a PC group, which is stabilized in a hydrophilic area of the active site. The phosphate group of PC forms hydrogen bonds with Arg218 and Gln211, while the positively charged nitrogen of the choline group participates in electrostatic interactions with Glu214. The carbonyl group of the *sn*-2 position interacts with the backbone amide of the oxyanion hole (Leu153 and Phe274) and with the catalytic serine through hydrogen bonding. The ether *sn*-1 oxygen atom in PAF analogs and the *sn*-1 oxygen atom in the carbonyl group in oxidized PCs forms a hydrogen bond with Gln352. Lp-PLA_2_ did not show enzymatic activity toward phospholipids containing long-chain fatty acids at the *sn*-2 position (Fig. 3). The MD simulation of the enzyme in the presence of PAF showed a dipalmitoyl phosphatidylcholine (DPPC) (colored in magenta) competing with PAF (colored in black) for the active site of the enzyme (Movie S8). DPPC initially binds to the active site, but soon after, it is displaced allowing PAF to occupy the active site. Lp-PLA_2_ contains a small pocket that accommodates short acyl-chain fatty acids esterified at the *sn*-2 position, and thus phospholipids with long-chain fatty acids at this position are excluded and cannot serve as substrates for this enzyme. The *sn*-2 pocket consists of residues Ala155, Leu159, Ala355, His151, Tyr160, and His272. PAF analogs containing a fatty acid with two and four carbon atoms at the *sn*-2 position are substrates for Lp-PLA_2_ because they fit into the small *sn*-2 pocket (Fig. 4*A* and Movies S9 and S10). PAF analogs with an *sn*-2 fatty acid containing 18 and 20 carbon atoms are not substrates for Lp-PLA_2_ because they do not fit into the small *sn*-2 pocket. The MD simulation of the PAF analog containing an azelaoyl (9:0, COOH) at the *sn*-2 position showed hydrogen bonding of the carboxylate group with residues His151, Tyr160, and the backbone amide of His272 (Fig. 4*A* and Movie S11).

Lp-PLA_2_ exhibited higher enzymatic activity toward oxidized PCs compared to PAF analogs (Fig. 3). It is worth mentioning that the PC analog of PAF-azelaoyl (9:0, COOH) is a ∼20-fold better substrate for Lp-PLA_2_. In fact, the azelaoyl-PC is the best substrate among the substrates tested in our assay (Fig. 3). The binding of azelaoyl-PC in the active site of Lp-PLA_2_ was similar to the binding of PAF-azelaoyl, showing the same extra hydrogen bond with the side chain of His272, compared to the other substrates (Fig. 4*B* and Movie S12). In addition, the oxygen atom of the *sn*-1 carbonyl group in the azelaoyl-PC forms a more frequent hydrogen bond with Gln352 since its geometry allows more exposure to the side chain of Gln352 compared with the ether oxygen of the PAF-azelaoyl (*SI Appendix*, Table S2 and Movies S11 and S12).

The same hydrogen bond was observed more frequently in all the oxidized PCs in contrast to the PAF analogs contributing to greater enzyme–substrate stability and explaining the optimum activity of Lp-PLA_2_ toward the oxidized PCs (*SI Appendix*, Table S2). Note that the terms “oxidized lipids or oxidized PCs” is generally used to include “oxygenated” polyunsaturated fatty acids (PUFAs) that are enzymatically produced as well as “oxidized lipids” that are produced via free radical oxidative mechanisms (like isoprostanes). PUFAs can be enzymatically oxygenated in their free form and then be incorporated into lysophospholipids by acyl transferases or in some special cases, such as with 15-lipoxygenase, the oxygenation can occur enzymatically on the intact phospholipid (30). The second most optimum substrate for Lp-PLA_2_ is the PC containing a succinoyl [5:0 (COOH)] at the *sn*-2 position (Fig. 3). The carboxylate group of the succinoyl-PC forms two hydrogen bonds with Tyr160 and His272 (Fig. 4*B* and Movie S13). A hydrogen bond with His151 was not observed in the MD simulation of succinoyl-PC compared to the azelaoyl-PC. Similar enzymatic activity with succinoyl-PC was observed toward the substrate containing a 5′-oxo-valeroyl [5:0 (ALDO)] at the *sn*-2 position. The oxygen atom of the aldehyde carbonyl group of this substrate also showed two hydrogen bonds with Try160 and His272, but no hydrogen bonding with His151 was observed (Fig. 4*B* and Movie S14). Finally, the oxidized PC containing a 9'-oxo-nonanoyl [9:0 (ALDO)] at the *sn*-2 position is a less optimum PC substrate for Lp-PLA_2_ but still better than all the PAF analogs (Fig. 3). The MD simulation of this substrate showed that the oxygen atom of the aldehyde carbonyl group participates only in one hydrogen bond with His151 (Fig. 4*B* and Movie S15). According to the literature, phospholipids containing F2-isoprostanes at the *sn*-2 position are substrates for Lp-PLA_2_ (10). Unfortunately, such phospholipids are not commercially available to be tested in our enzymatic assay. However, in an effort to have a complete picture of the Lp-PLA_2_ substrate specificity, an MD simulation of a PC containing F2-isoprostane at the *sn*-2 position was performed. This simulation showed that the *sn*-2 chain of F2-isoprostane-PC was accommodated in the small *sn*-2 pocket of Lp-PLA_2_, with its hydroxyl groups forming hydrogen bonding with residues Tyr160, His151, and His272 (Movie S16).

### Identification of the Headgroup Binding Pocket.

The MD simulation of Lp-PLA_2_ in the presence of inhibitor SB-402565 and various substrates revealed a binding pocket consisting of residues Arg218, Gln211, and Glu214, where the headgroup of the phospholipid binds. A similar binding pocket was identified in the other three major types of PLA_2_ enzymes including iPLA_2_ (Lys489, Asn658, and Asp733), cPLA_2_ (Arg200, Asn555, and Glu418), and sPLA_2_ (Arg62, Ca^2+^, and Glu55) (2, 21). In these three other enzymes, this pocket contains a positively charged residue, either a lysine or an arginine, that interacts with the phosphate group, an asparagine, a glutamine, or a calcium ion that also interacts with the phosphate group, and a negatively charged residue, either an aspartic or a glutamic acid, that interacts with the positively charged nitrogen atom of the choline group. While binding of a positively charged lysine or arginine to the phosphate group of c, i, and sPLA_2_s has been recognized and validated, this pocket was not previously identified and defined for Lp-PLA_2_ because the published crystallographic structures of the enzyme do not contain an inhibitor or substrate analog that interacts with this pocket. For example, Darapladib (*SI Appendix*, Figs. S3 and S7), for which a crystal structure is available (18), does not contain the extra methoxy-dihydropyrimidine substitution at the pyrimidone ring, which interacts with Arg218 and Gln211 (Fig. 2*B*). In addition, there are no available crystal structures of Lp-PLA_2_ with substrates or direct substrate analogs to determine the headgroup binding pocket. To experimentally confirm this binding pocket, the Lp-PLA_2_ mutants Q211A, E214A, and R218A were expressed and purified. The enzymatic activity of these three mutants toward PAF was lower than the wild-type (WT) Lp-PLA_2_, confirming the importance of Arg218, Glu214, and Gln211 for substrate binding (*SI Appendix*, Fig. S9).

## Discussion

**Fig. 5.**
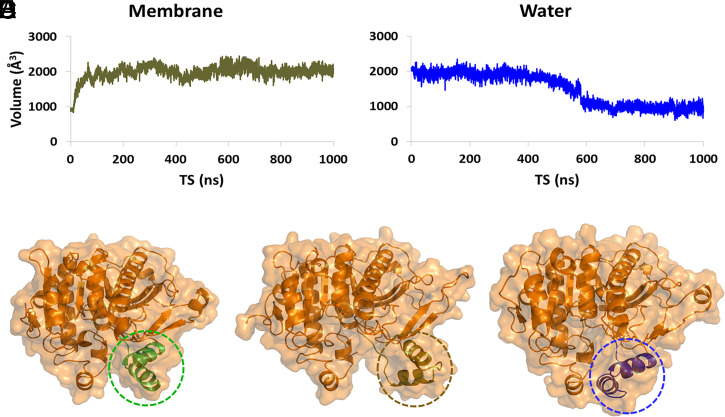
Allosteric regulation of Lp-PLA_2_ by the membrane bilayer (Movie S17). (*A*) Volume of the Lp-PLA_2_ active site during the MD simulation in the presence of a membrane patch, (*B*) volume of the Lp-PLA_2_ active site during the simulation in water, (*C*) conformation of Lp-PLA_2_ as described in the crystal structure (PDB ID: 3D59) used for the MD simulation (circled residues 100 to 130 are colored in green, (*D*) conformation of Lp-PLA_2_ after the MD simulation in the presence of a membrane patch (circled residues 100 to 130 are colored in brown), and (*E*) conformation of Lp-PLA_2_ after the MD simulation in presence of water (circled residues 100 to 130 are colored in blue).

PLA_2_ enzymes are water soluble and act at the interface between the aqueous phase and the membrane bilayer, micelle, or the lipoprotein phospholipid monolayer. Thus, they contain an interfacial surface with amphipathic properties that facilitates their association with the membrane to extract and bind their phospholipid substrate. Our group introduced the concept that upon association, membranes allosterically regulate PLA_2_ enzymes to adopt an “open” conformation, enabling them to extract and bind their substrate (2, 21). Lp-PLA_2_ is another example of an enzyme that undergoes allosteric regulation by membranes. During the MD simulation of the Lp-PLA_2_ crystal structure without an inhibitor or substrate in the active site, but in the presence of a membrane patch, the volume of the active site was increased from ∼900 to 2,000 Å^3^ (Fig. 5*A*). The peptide region 100 to 130, which consists of two α-helices connected with a loop, was found to be solely responsible for controlling the active site volume of Lp-PLA_2_. Part of this peptide region was also found to mediate interaction with membranes in HD-XMS studies (Fig. 1) (19, 20). The MD simulation showed that this region is flexible, causing a conformational change that allows the enzyme to shift from a “closed” conformation (Fig. 5*C* and Movie S17) to an “open” conformation (Fig. 5*D* and Movie S17). To further ratify our observation, the “open” conformation of the enzyme was placed in a water box and subjected to 1-μs simulation. This simulation showed that in the presence of water, the conformation of the enzyme shifts to “closed” because the peptide region 100 to 130 moves back to its initial position (Fig. 5*E* and Movie S17). In addition, the volume of the active site was decreased from 2,000 to 900 Å^3^ (Fig. 5*B*).

To experimentally confirm the conformational change involving peptide region 100 to 130, a site-directed tryptophan fluorescence experiment was conducted to detect the environmental changes of tryptophan residues (31). The Lp-PLA_2_ sequence was found to contain seven tryptophan residues. Among them, Trp105 and Trp115 are located in the region containing the two helices which exhibits the conformational change, while Trp97, Trp105, Trp115, Trp134, Trp203, Trp298, and Trp405 are located in other regions, (*SI Appendix*, Fig. S10*A*). All tryptophan residues except for Trp105 and Trp115 were replaced with phenylalanine residues, and that allowed us to observe the environmental change of this region upon membrane binding. The enzymatic activity of Trp-less Lp-PLA_2_ toward PAF was lower than that of WT, which is consistent with the activity of the W298A mutant (*SI Appendix*, Figs. S2*C* and S10*B*), and we could confirm that the mutant is still active. Then, we measured the emission spectra of the Trp-Less Lp-PLA_2_ in the presence or absence of 1,2-dimyristoyl-sn-glycero-3-phosphocholine (DMPC) small unilamellar vesicles. The tryptophan fluorescence showed a redshift in the presence of lipid vesicles, and the maximum emission wavelength was significantly longer than in the absence of the vesicles (*SI Appendix*, Fig. S10*C*). This result indicates that the peptide region 100 to 130 is exposed to a more polar environment by the conformational change from “closed” to “open” upon binding of the enzyme to lipid vesicles. In other words, this region is indeed involved in a conformational change that occurs upon membrane binding, as illustrated in Movie S17.

**Fig. 6.**
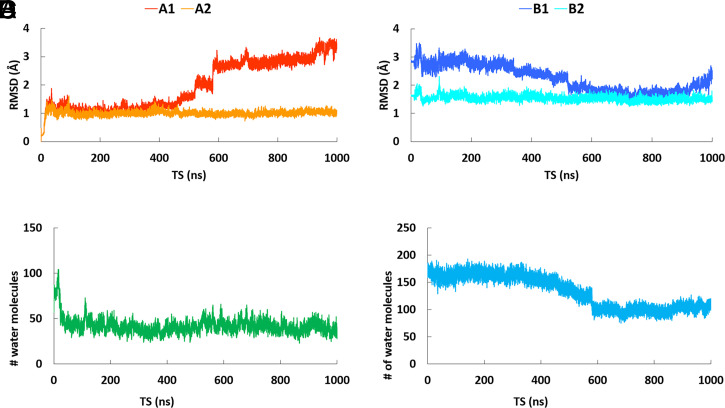
RMSD versus time of the simulation for the “open” conformation of Lp-PLA_2_ in the presence of water. (*A*, *A1*) The backbone atoms of Lp-PLA_2_ in the trajectory were aligned to the starting (“open”) conformation of the simulation (red color), (*A*, *A2*) same alignment as *A*1, but the peptide region 100 to 130 was excluded (orange color), (*B*, *B1*) The backbone atoms of Lp-PLA_2_ in the trajectory were aligned to the crystallographic conformation (PDB ID: 3D59, blue color), (*B*, *B2*) same alignment as *B1*, but the peptide region 100 to 130 was excluded (cyan color). (*C*) Number of water molecules in the active site of Lp-PLA_2_ versus time of the simulation in the presence of a membrane patch, and (*D*) number of water molecules in the active site of Lp-PLA_2_ versus time of the simulation in the presence of water.

RMSD analysis revealed that the peptide region 100 to 130 is solely responsible for causing the conformation changes upon membrane association and for controlling the volume of the Lp-PLA_2_ active site (Fig. 6*A*). Fig. 6 *A*, *A1* shows the RMSD versus time of simulations for the MD simulation of the “open” conformation of Lp-PLA_2_ in the presence of water. It is remarkable that between 500 and 600 ns, the RMSD value changes drastically from ∼1.5 to 2.5 Å, indicating the beginning of a conformational change. At the end of the simulation, the RMSD value was stabilized at ∼3.4 Å. When the peptide region 100 to 130 was excluded from the backbone atom alignment, the rest of the enzyme backbone showed no changes in the RMSD value (Fig. 6 *A*, *A2*). This is indicative that this peptide region is solely responsible for the conformational changes of Lp-PLA_2_. The backbone atoms of the “open” conformation of Lp-PLA_2_ in the presence of water were also compared to the backbone atoms of the crystallographic conformation (Fig. 6*B*). This RMSD analysis showed that while the simulation progresses and the conformation shifts from “open” to “closed,” between 500 and 600 ns, the RMSD value changes from ∼2.5 to 1.7 Å, indicating that the “closed” conformation is closer to the crystallographic conformation (Fig. 6 *B*, *B1*). At completion of the simulation, the RMSD value was stabilized at 2.4 Å because the peptide region 100 to 130 closes completely to protect the hydrophobic region of the active site from water (Fig. 5*E*). When the peptide region 100 to 130 was also excluded from the backbone atom alignment, the rest of the enzyme backbone showed no changes in the RMSD value as expected (Fig. 6 *B*, *B1*). Movie S17 shows the opening (peptide region 100 to 130 is colored in dark green) and the closing (peptide region 100 to 130 is colored in blue) of the Lp-PLA_2_ active site during the MD simulations in the presence of a membrane patch and water, respectively.

Another interesting point is the amount of water in the active site of Lp-PLA_2_ during the MD simulation in the presence of the membrane (Fig. 6*C*) and water (Fig. 6*D*). The presence of the membrane creates an optimum environment for the hydrophobic pocket of the active site, with a low number of water molecules (∼50), allowing the enzyme to adopt an “open” conformation and extract its substrate (Fig. 6*C*). In the presence of water, the “open” conformation of the enzyme contains ∼170 water molecules in the active site. Between 500 and 600 ns, when the “open” conformation shifts to “closed, ” the Lp-PLA_2_ active site expels water molecules to protect the hydrophobic pocket of the active site. At completion of the simulation, the Lp-PLA_2_ active site contains ∼100 water molecules (Fig. 6*D*). *SI Appendix*, Fig. S11 depicts the RMSD versus the time of the simulation for the MD simulations performed on SB-402564 inhibitor, the PAF analogs, and the oxidized PCs. The Lp-PLA_2_ backbone alignment for the simulations performed on BM1-3, did not show significant changes of the RMSD values during the time course of each simulation (*SI Appendix*, Fig. S11*A*). In contrast, the simulations performed on BM4-6 showed significant changes of the RMSD values during the time course of the simulation (*SI Appendix*, Fig. S11*B*). The enzyme-inhibitor complexes for BM1-3 were generated using the crystallographic conformation of Lp-PLA_2_, which is closer to the “closed” conformation of Lp-PLA_2_. The enzyme-inhibitor complexes for BM4-6 were generated using the “open” conformation of Lp-PLA_2_. During the simulations on BM4-6, the peptide region 100 to 130 moves closer and interacts with the inhibitor shifting the Lp-PLA_2_ conformation to “closed.” The Lp-PLA_2_ backbone alignment for the MD simulations in the presence of substrates showed significant changes of the RMSD values during the time course of each simulation (*SI Appendix*, Fig. S11 *C* and *D*). In the case of the substrates, Lp-PLA2 adopts an “open” conformation on the surface of the membrane to extract and bind its phospholipid substrate.

## Conclusion

Lp-PLA_2_ represents a unique paradigm for allosteric regulation by membranes that is distinct compared to the allosteric regulation of cPLA_2_, iPLA_2_, and sPLA_2_ (2, 21). Our studies support the existence of allosteric sites located at the interfacial surface of PLA_2_ enzymes where the membrane binds, causing a conformational change that shifts the Lp-PLA_2_ conformation from “closed” to “open” (Movie S17). Using state-of-the-art experimental and computational techniques, the binding interactions of the SB-402564 inhibitor with the active site of the enzyme were determined. Extensive MD simulations revealed that the inhibitor keeps the conformation of the Lp-PLA_2_ “closed” by interacting with residues located in the peptide region 100 to 130. Substrate specificity was also determined for PAF analogs and oxidized PCs using our liquid chromatography mass spectrometric assay. MD simulations allowed us to identify a small *sn*-2 pocket where the *sn*-2 fatty acid binds. Azelaoyl-PC (*sn*-2 chain 9:0 COOH) is the best substrate for Lp-PLA_2_ because its carboxylate group forms hydrogen bonding with His151, Tyr160, and His272, which are residues in the small *sn*-2 pocket. Finally, a headgroup pocket consisting of residues Arg218, Gln211, and Glu214 was identified using MD simulations and confirmed using site-directed mutagenesis. *SI Appendix*, Table S3 provides a list of the movies and their subject matter.

## Supplementary Material

Supplementary File

Supplementary File

Supplementary File

Supplementary File

Supplementary File

Supplementary File

Supplementary File

Supplementary File

Supplementary File

Supplementary File

Supplementary File

Supplementary File

Supplementary File

Supplementary File

Supplementary File

Supplementary File

Supplementary File

Supplementary File

## Data Availability

All  study data are included in the article and/or supporting information.
