# Supplementary material for: Lipoprotein-associated phospholipase A2: A paradigm for allosteric regulation by membranes
Source: Proc Natl Acad Sci U S A. 2022 Jan 7;119(2):e2102953118. doi: 10.1073/pnas.2102953118 (PMC8764669; doi:10.1073/pnas.2102953118)
Supplement: Supplementary File [file pnas.2102953118.sapp.pdf]

Lipoprotein-associated phospholipase A_2: A paradigm for allosteric regulation by membranes

Varnavas D. Mouchlis*, Daiki Hayashi, Alexis M. Vasquez, Jian Cao, J. Andrew McCammon, and Edward A. Dennis*

Department of Chemistry and Biochemistry and Department of Pharmacology, School of Medicine, University of California, San Diego, La Jolla, CA 92093-0601, USA

*Corresponding Authors: Varnavas D. Mouchlis, email: vmouchlis@gmail.com; Edward A. Dennis, email: edennis@ucsd.edu, phone: +1 858 534 3055.

Table of Contents

Experimental and Computational Methods 2
Protein expression and purification 2
Site-directed mutagenesis for single mutants 2
Enzymatic activity 2
LC/MS Assay 2
Mass spectrometry 3
Preparation of samples for H/D exchange 3
Proteolysis and liquid chromatographic mass spectrometric (LC/MS) analysis 3
Construction and purification of the W97/W134/W203/W298/W405F (Trp-less) mutant 3
Preparation of DMPC small unilamellar vesicles 4
Tryptophan fluorescence measurement 4
Docking protocol 4
Molecular dynamics (MD) simulations 4
Supplementary Figures 7
Figure S1 7
Figure S2 8
Figure S3 9
Figure S4 10
Figure S5 11
Figure S6 12
Figure S7 13
Figure S8 14
Figure S9 15
Figure S10 16
Figure S11 17
Supplementary Tables 18
Table S1 18
Table S2 18
Table S3 18
References 20

Experimental and Computational Methods

Protein expression and purification. Human recombinant wild-type Lp-PLA_2 (residues 47-441) was expressed using BL21 (DE3) cells as previously described (1, 2). Briefly, 1 L of cell culture in LB media was incubated at 37 ^oC in a shaker flask for 3 h. The culture was induced with 0.5 mM IPTG for an additional 4 h and the cells were harvested by centrifugation at 5,000 rpm for 10 min at 4 ^oC. The cell pellet was suspended in PBS buffer containing 10 mM CHAPS, 1 mM DTT, and 0.01% lysozyme. Protein extracts were obtained after 30 min incubation at room temperature in PBS buffer. The lysates were briefly sonicated and centrifuged at 20, 000 g for 30 min at 4 ^oC. The GST-Lp-PLA_2 fusion protein was purified by GST resin (GenScript). The GST tag was cleaved from the fusion protein via on-column cleavage using enterokinase (GenScript) and Lp-PLA_2 was eluted with 20 mM Tris, 150 mM NaCl, 10% glycerol, pH = 7.5, 1 mM DTT. His-tag enterokinase was removed from purified Lp-PLA_2 eluates using a Ni-NTA column (Qiagen). Purified Lp-PLA_2 was concentrated using Amicon Ultra-15 (Millipore).

Site-directed mutagenesis for single mutants. Recombinant Lp-PLA_2 H151A, L153A, F156A, F274A, W298A, and Q352A mutants were created using the QuickChange® Site-Directed Mutagenesis Kit. Primers were obtained from Allele Biotechnology. The Lp-PLA_2 Q211A, E214A, and R218A mutants were constructed by the long PCR method. The mutations were confirmed by DNA sequencing. The recombinant Lp-PLA_2 mutants were expressed and purified according to a previously published method (1). Briefly, the plasmid was transfected into BL21 (DE-3) *E. Coli* and expression was induced with 0.5 mM IPTG for 4 h at 25°C. The recombinant enzyme was purified with glutathione Sepharose, and GST tag was cleaved by enterokinase.

Enzymatic activity. Enzymatic activity of recombinant wild-type and mutant Lp-PLA_2 was measured using a previously descripted radioactive assay (1, 2). Briefly, Lp-PLA_2 substrate was created using 100 μM [3H-acteyl] PAF in 100 mM HEPES buffer (pH = 7.5) containing 1 mM EGTA, 1 mM DTT, 2 mM CHAPS. The enzymatic reaction took place in 500 μL of substrate for 20 min at 37 ^oC. The reaction was quenched using 500 μL of 10M acetic acid. The released 3H-acetate was separated by C18 reversed-phase cartridges (Phenomenex) and quantitated by scintillation counting.

LC/MS Assay. The LC/MS assay to define substrate specificity was performed as previously described (3, 4). Briefly, pure recombinant Lp-PLA_2 enzyme was incubated with mixed micelle substrate consisting of 100 μM phospholipid, 400 μM C12E8 surfactant, and 2.5 μM 17:0 lysophospholipid as an internal standard for 30 min at 40°C. The reaction was quenched with methanol/acetonitrile (80/20). The lysophospholipid product of the reaction was quantified using

HILIC column in an HPLC system and MRM analysis using an AB Sciex 4000 QTRAP triple quadrupole MS system.

Mass Spectrometry. The lyso-species were detected in positive electrospray ionization (ESI) mode. Molecular species were detected as $[M+H]^+$ ions in positive ion mode. Curtain gas (CUR), nebulizer gas (GS1), and turbo-gas (GS2) were set to 10 psi, 50 psi, and 20 psi, respectively. The electrospray voltage was set to +4.5 kV, and the turbo ion spray source temperature was set to 500 °C. The lyso-species were analyzed using scheduled multiple reaction monitoring (MRM). Declustering potentials and collision energies were optimized for each analyte to achieve optimal mass spectrometric detection. Nitrogen was employed as the collision gas. Data acquisitions were performed using Analyst software. MultiQuant software was used to quantify all metabolites.

Preparation of samples for H/D exchange. Deuterium oxide (D_2O) buffer containing 8.3 mM Tris-HCl (pH 7.5) and 50 mM NaCl was used as previously described (1, 2). Briefly, 14 µL of Lp-PLA_2 (total 48 µg of enzyme) were mixed with 42 µL of D_2O buffer to a final concentration of 75% D_2O. Inhibitor binding experiments were performed by initially incubating Lp-PLA_2 with 875 µM of GSK-SB402564 in DMSO (protein/inhibitor ratio of 1:5) for 30 minutes at room temperature. An equivalent amount of DMSO was added to the control experiments. D_2O buffer was added to the samples, and they were incubated for an additional 10, 30, 100, 300, 1000, 3000, and 10,000 s. The final concentration of GSK-SB402564 after the addition of D_2O buffer was 200 µM. The H/D exchange was quenched by adding 18 µL of ice-cold quench solution containing 0.8% MGuHCl, and 0.08% formic acid (pH 2.5). The samples were immediately frozen on dry ice and were stored at -80 ^oC until analysis (within 48 h).

Proteolysis and liquid chromatographic mass spectrometric (LC/MS) analysis. For proteolysis and the mass spectrometry analysis, the samples were thawed on ice and injected through a pepsin column for digestion as described previously (1, 2). The peptide fragments were separated with a C18 reverse phase analytical column at 0 ^oC. The peptides were then analyzed on an OrbiTrap Elite Mass Spectrometer (ThermoFisher Scientific, San Jose, CA). Tandem mass spectrometry (MS/MS) data was analyzed with SEQUEST (Thermo Finnigan, Inc.) software. DXMS Explorer was employed for analysis of the results and to calculate the average of mass envelope.

Construction and purification of the W97/W134/W203/W298/W405F (Trp-less) mutant. W97, W134, W203, W298, W405 in Lp-PLA_2 were all substituted with phenylalanine using the Phusion Site-Directed Mutagenesis Kit. The initial template was N-terminal GST tagged human Lp-PLA_2 encoding pGS-21a plasmid (1). The mutations were confirmed by DNA sequencing. Trp-less Lp-PLA_2 was subcloned into a pGEX-6P vector containing a 6XHis tag at the C-terminal. The plasmid

was transfected into BL21 (DE-3) E. Coli and expression was induced with 0.4 mM IPTG for 4 h at 25°C. The recombinant enzyme was purified with glutathione Sepharose, and GST tag was cleaved by PreScission protease. After the cleavage of the GST tag, the protein was further purified using Ni-NTA Sepharose. The purified protein was dialyzed against the buffer (PBS, 1 mM DTT, 20% Glycerol). The purity of the protein was analyzed by Coomassie brilliant blue staining and was greater than 90%.

Preparation of DMPC small unilamellar vesicles. DMPC (10 mg) was dried using argon gas and lyophilized for 1 hr. The DMPC sample was then vortexed in 1 mL of PBS buffer to form lipid vesicles. The suspensions were extruded through 0.2 µm pore size filter using the extruder by Avanti Polar Lipids. The solution was centrifuged at 10,000 × g for 10 min to remove the large vesicles.

Tryptophan fluorescence measurement. 3 µM of purified Trp-less Lp-PLA$_2$ was incubated in the PBS buffer which contained 60 µM DMPC small unilamellar vesicles for 10 min. The control experiment was performed under the same conditions without DMPC. After incubation, the Trp fluorescence emission spectra from 310 to 450 nm was measured with an excitation at 295 nm at room temperature.

Docking protocol. Multiple crystal structures of Lp-PLA$_2$ were downloaded from protein data bank and prepared using the Protein Preparation Wizard module implemented in Schrödinger suite (5). The active site of Lp-PLA$_2$ was computationally defined using SiteMap algorithm implemented in Schrödinger suite (6, 7). SiteMap identifies the possible binding pockets by utilizing a water probe to scan the entire surface of the protein for cavities that are accessible to solvent and small molecules. Residues Phe110, Leu121, Phe125, Leu153, Phe156, Tyr160, Gln211, Glu214, Arg218, His272, Ser273, Phe274, Asp296, Trp298, His351, Gln352, Phe357, Ile365 and Leu369 were chosen to define the active site of Lp-PLA$_2$ for docking calculations using a crystal structure with PDB ID 3D59 (8). The 3D structure of GSK-SB402564 inhibitor and phospholipid substrates were sketched and optimized using Maestro and LigPrep implemented in Schrödinger Suite (5). Rigid and flexible docking with was performed using Glide and Induced Fit modules both implemented in Schrödinger Suite (9-11). The Glide extra precision (XP) scoring function was employed for docking calculations (12).

Molecular dynamics (MD) simulations.

Enzyme-membrane complexes. The input files necessary for NAMD were generated using the Membrane Builder implemented in CHARMM-GUI (13, 14). The membrane patch consisted of POPC, SAPC, POPE, POPA, POPG, POPS, SAPI(4,5)P2 and cholesterol. The average ratios of the

lipids were chosen to be 0.65 for PC, 0.10 for PE, 0.10 for PI(4,5)P2, 0.06 for PS and 0.09 for PA and PG. The average cholesterol/lipid ratio was chosen to be 0.40. Lipid composition was chosen to contain high ratios of PC and cholesterol which are the most abundant phospholipid species in lipoproteins (15). Each system was solvated with TIP3P water molecules and neutralized with 150 mM sodium chloride using the Visual Molecular Dynamics (VMD) package (16).

Equilibration and production runs. Molecular dynamics simulations were carried out using NAMD 2.12 (17). The following minimization and equilibration protocol was performed: a minimization of 80,000 steps was initially performed by applying harmonic constraints on the enzyme-ligand-membrane that were gradually turned off using a constraint scaling factor, followed be a second 120,000 steps minimization without constraints. An initial equilibration of 10,000 steps was performed by also applying harmonic constraints on the enzyme-ligand-membrane that were gradually turned off using the same constraint scaling factor followed by a second 10,000 steps equilibration without constraints. During the equilibration, each system was slowly heated and held to 310 K using temperature reassignment with reassignment frequency of 500 timesteps (1000 fs) and a reassignment increment of 1 K. The above minimization and equilibration protocol were sufficient to induce the appropriate disorder of a fluid-like bilayer, avoid unnatural atomistic positions, and failure of the simulations by atoms moving at extremely high velocities. Each system was finally subjected to a 1 μs production run. For each production run, the temperature was maintained at 310 K using the Langevin thermostat with Langevin coupling coefficient of 1/ps (18). The *NPT* ensemble was employed and the pressure was kept constant at 1.01325 kPa using the Langevin piston method with the "useGroupPressure", "useFlexibleCell" and "useConstantArea" parameters turned on (19). A time step of 2 fs was used in combination with the SHAKE algorithm to hold the bonds to hydrogen atoms similarly constrained (20). Nonbonded and full electrostatic interactions were calculated every 1 and 2 time steps, respectively. Switching functions are used to smoothly take electrostatic and van der Waals interactions to zero with switching distance 10 Å and cutoff 12 Å. Long-range electrostatic forces in the periodic system were evaluated using the Particle Mesh Ewald (PME) Sum method with grid spacing 1/Å (21). The CHARMM General Force Field (CGenFF) and the CHARMM36 all-atom additive force field and parameters were used for the simulations (22, 23).

Binding pocket volume calculations. The POVME algorithm was employed for calculating the volume of the binding pocket of each enzyme over the time of each simulation (24). A total number of 6,252 frames from each simulation trajectory were used for the calculations. The frames were aligned on the initial complex that was used to carry out the simulation using VMD and were saved in multi-frame PDB format. For defining the "inclusion sphere" that entirely encloses the binding pocket of each enzyme, the center of mass of the residues within 5 Å around the bound phospholipid molecule was used as the x, y, z coordinates of the sphere. An "inclusion

sphere" radius of 11 Å was used. Equidistant points were generated in POVME using a grid spacing of 1 Å and a distance cutoff of 1.09 Å.

Supplementary Figures

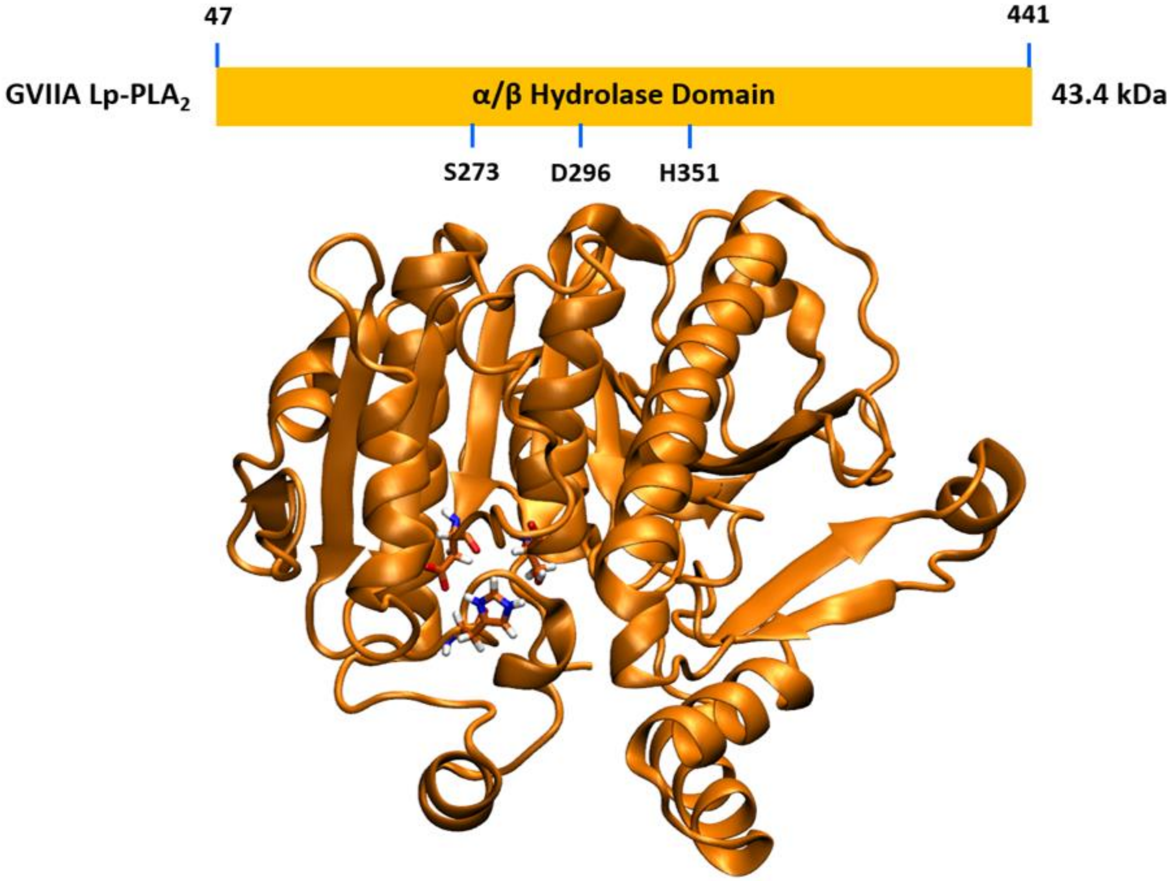

Figure S1. Three-dimensional structure of Lp-PLA_2 (PDB ID: 3D59).

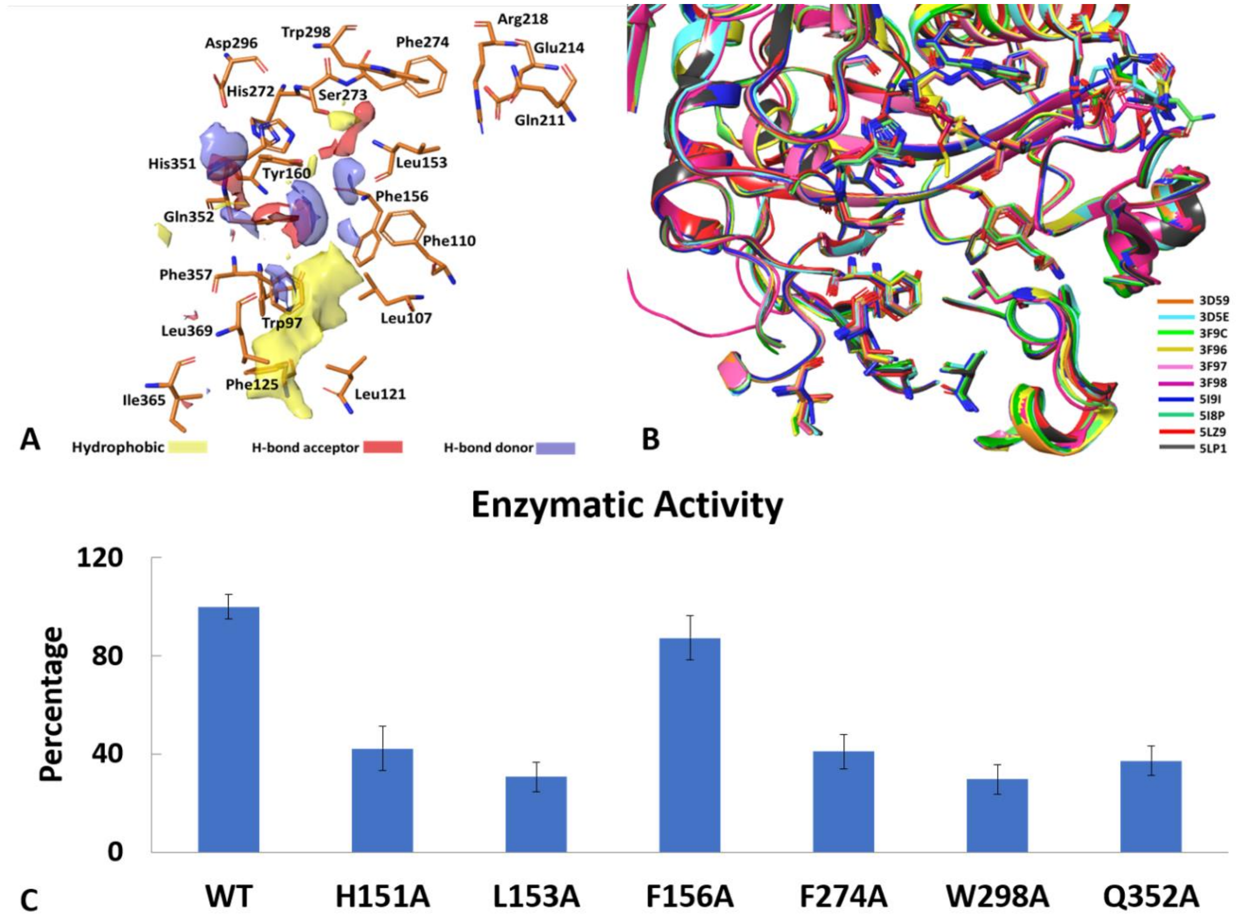

Figure S2. The active site of Lp-PLA_2. (A) Active site residues 5 Å around the hydrophobic, H-bond acceptor and donor SiteMap fields, (B) alignment of various Lp-PLA_2 crystal structures, and (C) enzymatic activity of recombinant Lp-PLA_2 mutants compared to wild-type.

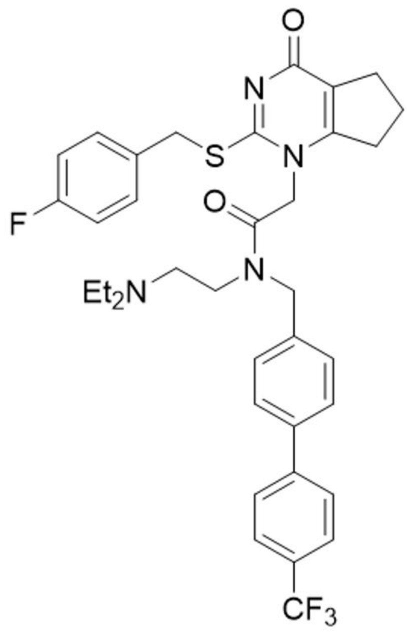

Darapladib

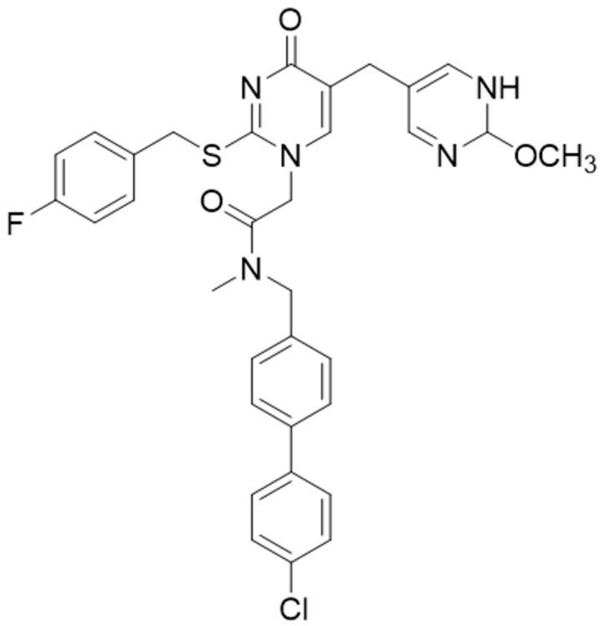

SB402564

Figure S3. Chemical structure of two potent Lp-PLA_2 inhibitors.

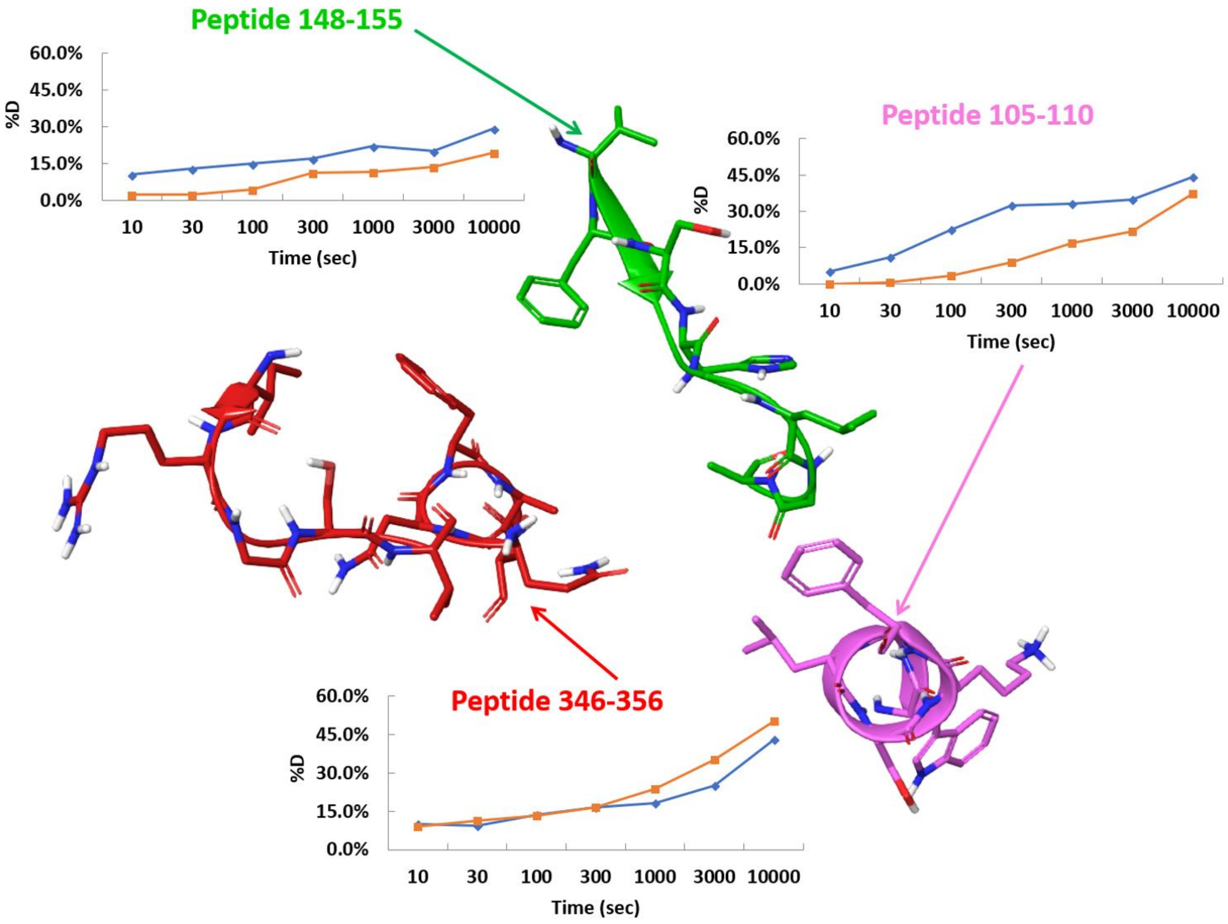

Figure S4. H/D exchange results upon binding of GSK-SB402564 to the Lp-PLA$_2$ active site. Percentage of deuteration level is presented for each peptide region of Lp-PLA$_2$ in the absence (blue) and presence (red) of GSK-SB402564. Three peptide regions showed changes of deuteration rates upon binding with GSK-SB402564: residues 105-110 (pink), 148-155 (green), 346-356 (red).

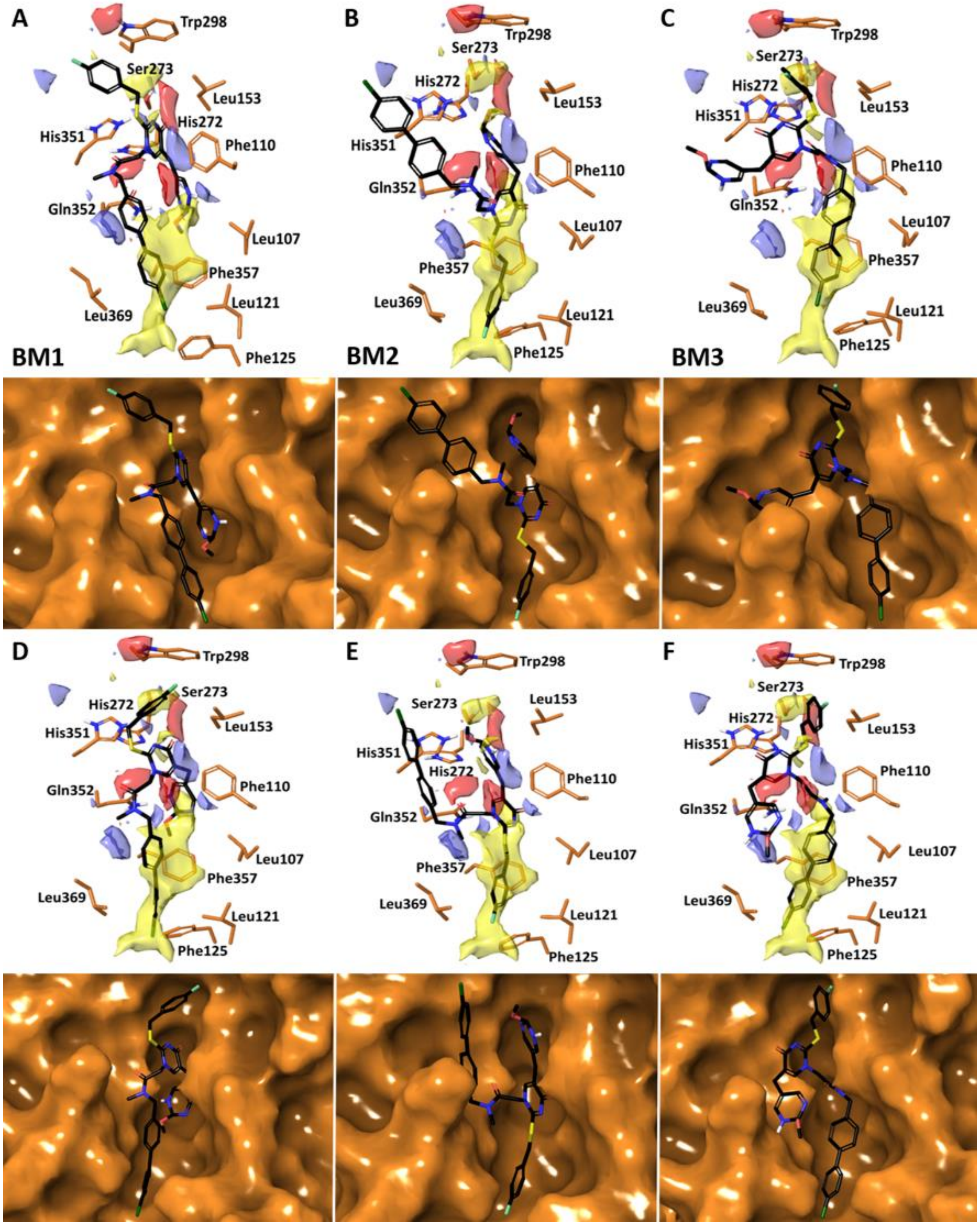

Figure S5. Binding modes of SB-402564 predicted by (A-C) rigid-receptor docking, and (D-F) induced fit-receptor docking.

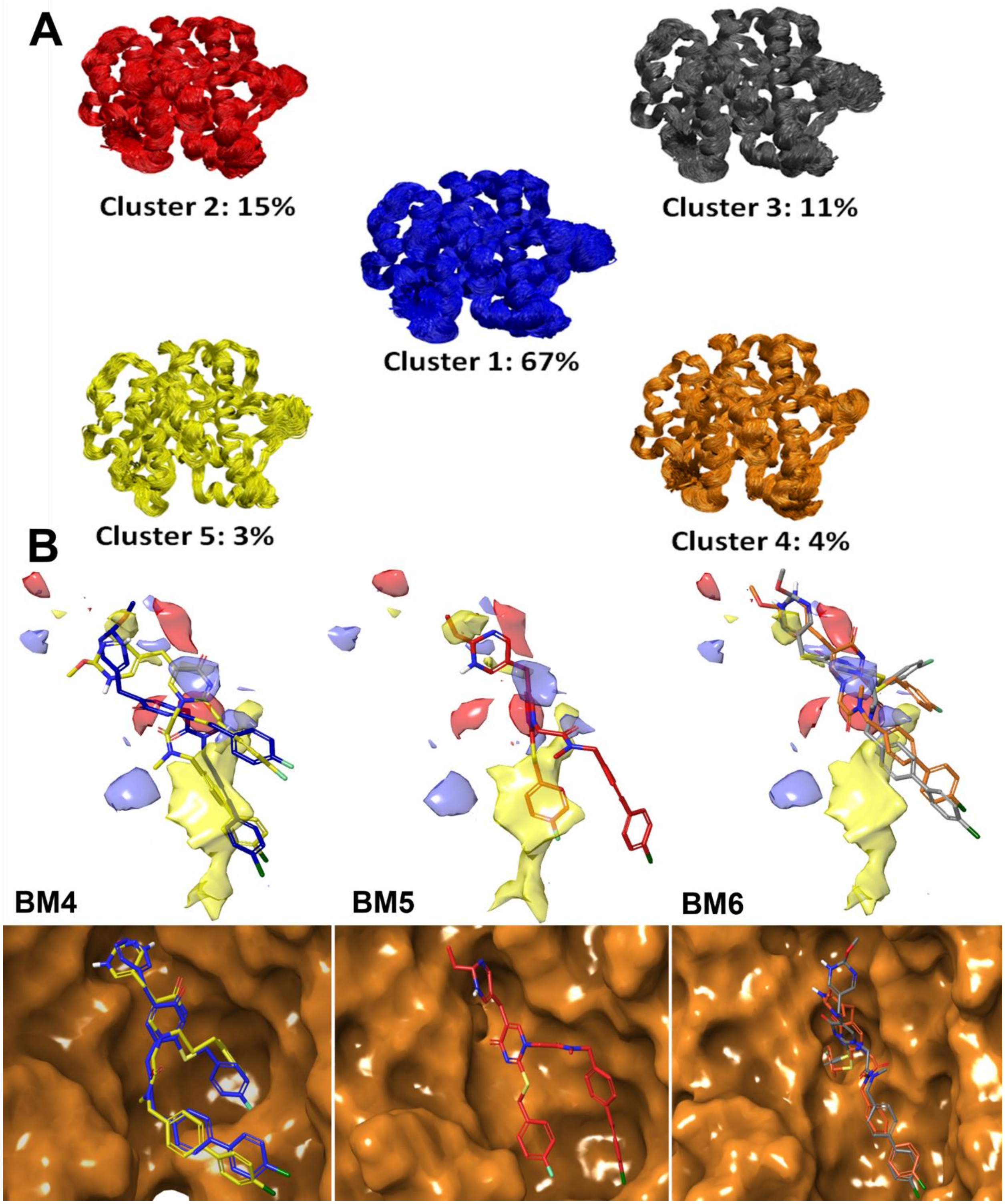

Figure S6. The relaxed complex scheme (RCS). (A) Clustering analysis allowed the identification of five Lp-PLA$_2$ conformations suitable for docking calculations, and (B) binding mode of SB-402564 in each cluster. The color of SB-402564 in each binding mode was selected to match the color of each conformational cluster.

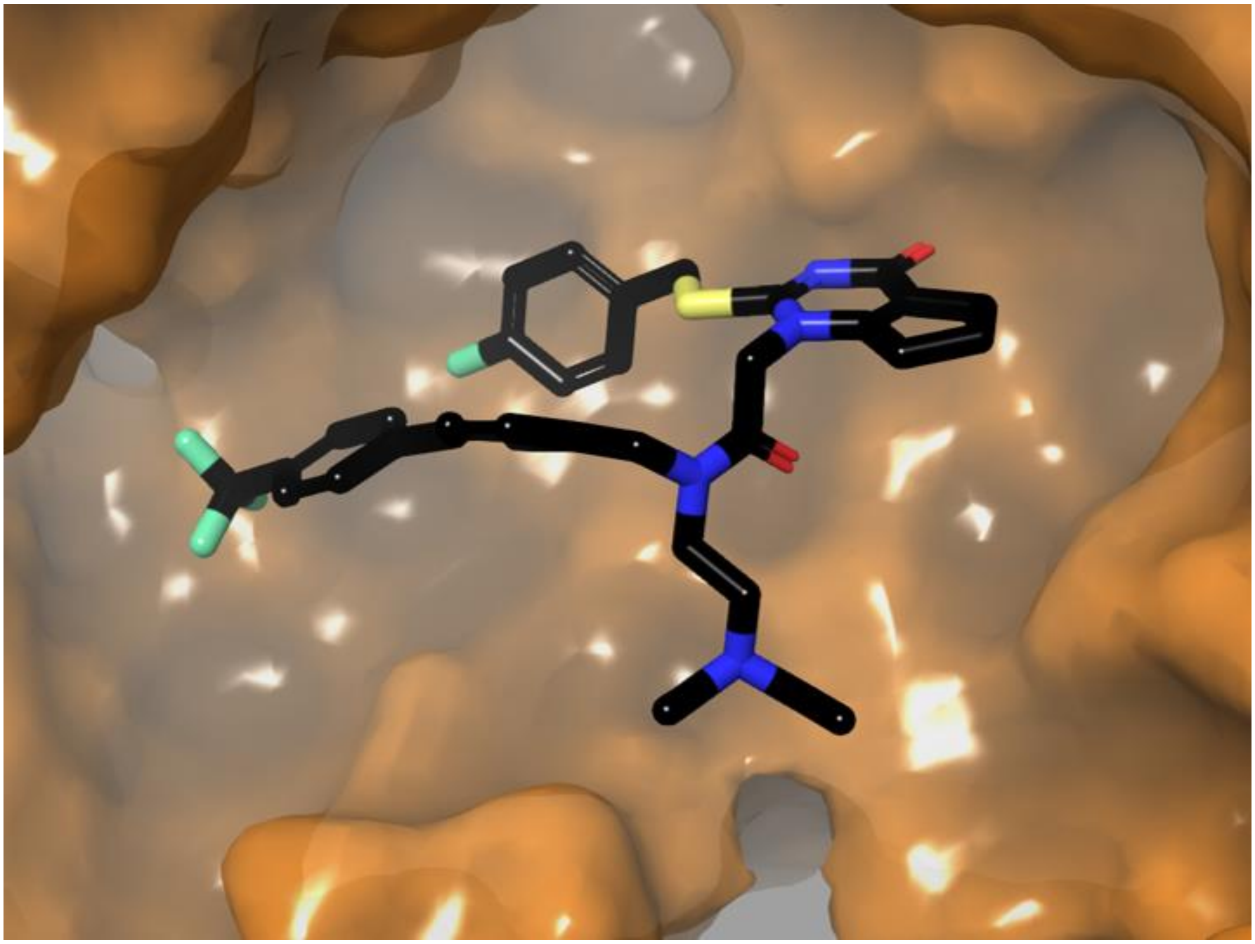

Figure S7. Conformation and orientation of Darapladib in the active site of Lp-PLA$_2$ according to the crystal structure (25).

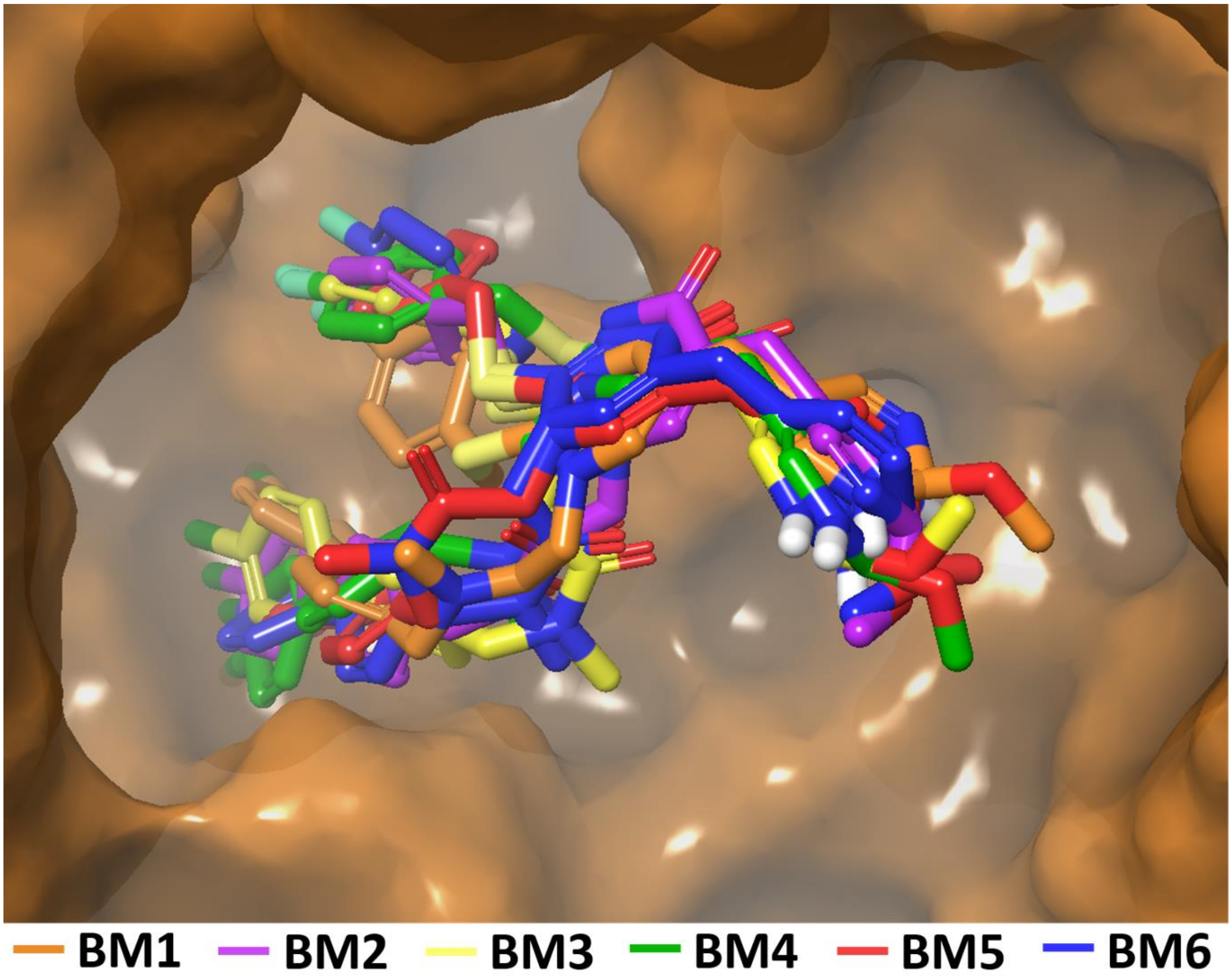

Figure S8. Binding mode of SB-402564 after redocking in the active site of Lp-PLA$_2$ using the optimized by MD simulations enzyme-inhibitor complex.

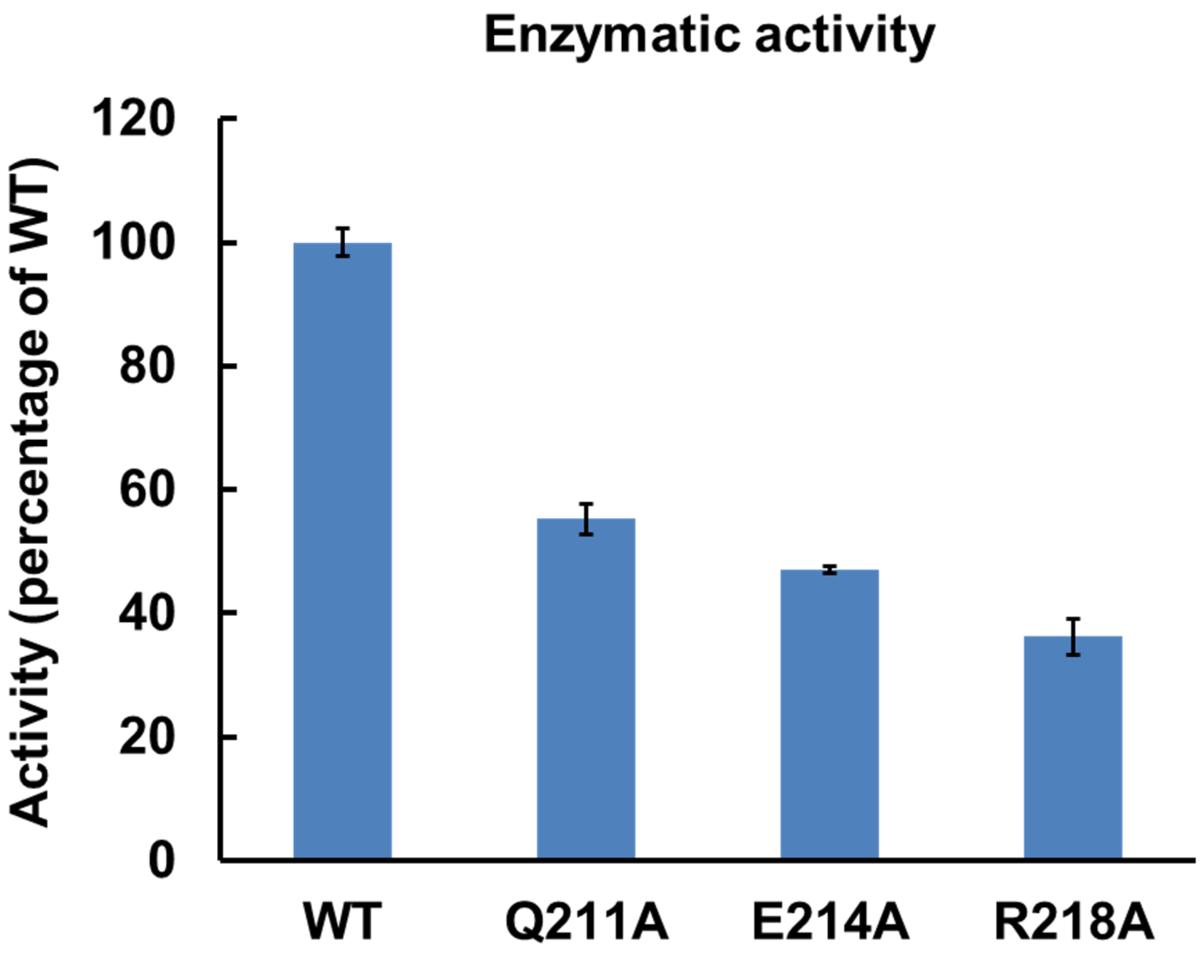

Figure S9. The Lower enzymatic activity of the recombinant Lp-PLA$_2$ Q211A, E214A, and R218A mutants compared to wild-type indicates that these three residues are important for the binding of the substrate headgroup.

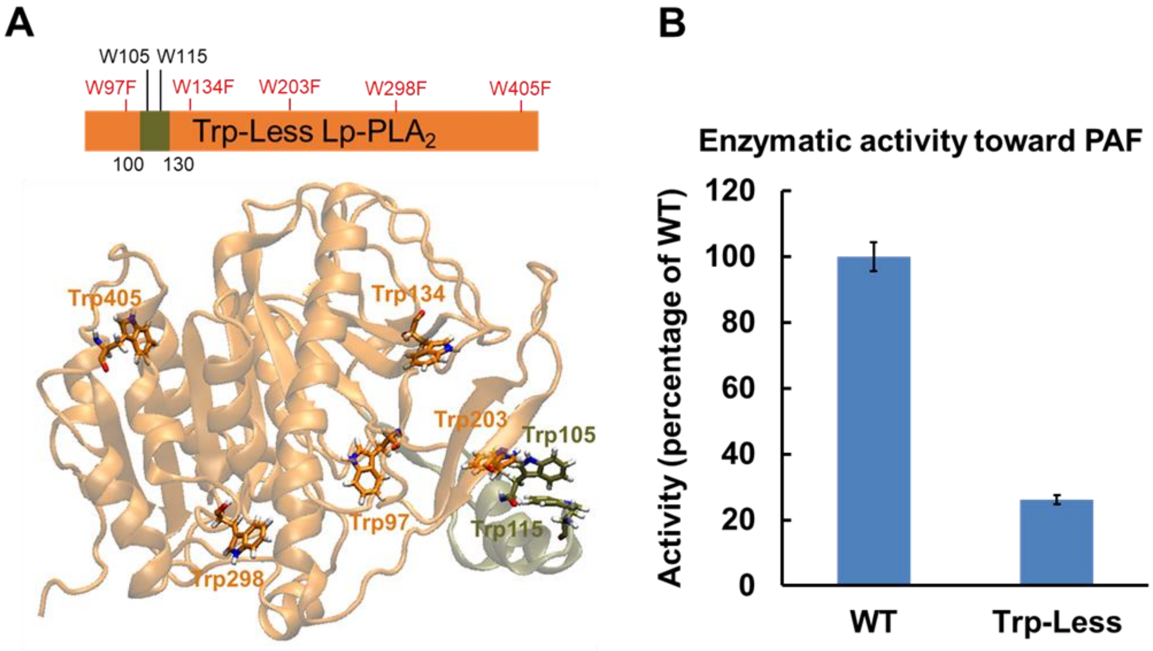

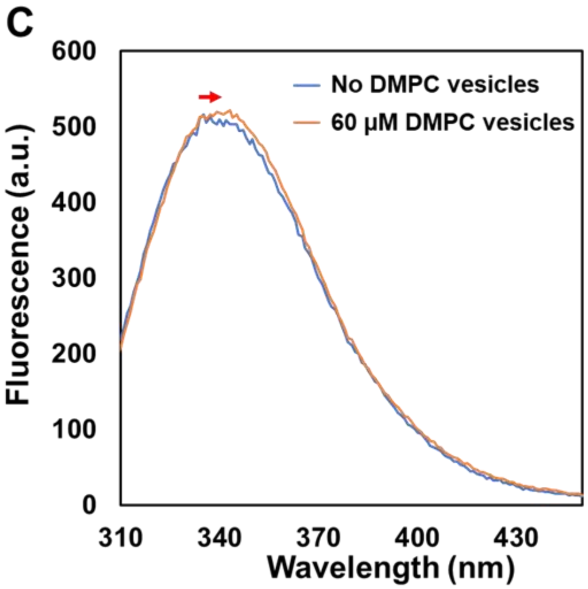

	Em_{max} (nm) *N=4*
No DMPC vesicles	337.30 ± 2.1
60 μM DMPC vesicles	341.04 ± 1.4*

Figure S10. Site-directed tryptophan fluorescence of Lp-PLA$_2$ containing two Trp in peptide region 100 to 130. (A) Localization of all tryptophan residues in human Lp-PLA$_2$ including the tryptophan residues that were mutated. (B) Enzymatic activity of Trp-less and WT Lp-PLA$_2$ toward PAF was measured using the LC-MS/MS assay. (C) The emission spectrum of Trp-less Lp-PLA$_2$ was measured in the presence or absence of 60 μM DMPC vesicles with an excitation at 295 nm and the maximum emission. The experiment was repeated four times using two different preparations of protein. The representative spectra are shown. Asterisk indicates statistical significance (*p<0.05, Student's *T*-test).

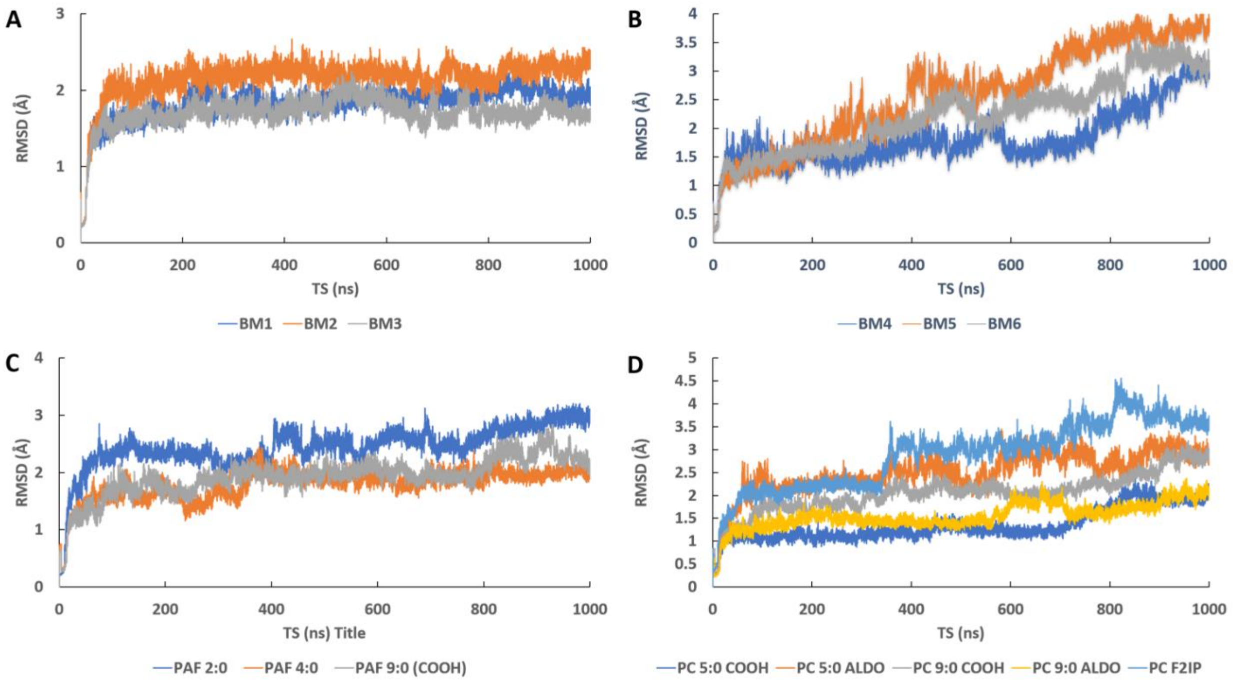

Figure S11. The root-mean-square deviation (RMSD) of the enzyme backbone atoms during the time course of the simulation for (A) BM1-3 for which the enzyme-inhibitor complex was generated using the crystallographic conformation of Lp-PLA$_2$, (B) BM4-6 for which the enzyme-inhibitor complex was generated using the "open" conformation of Lp-PLA$_2$, (C) MD simulations in the presence of each PAF analog, and (D) MD simulations in the presence of each oxidized PC.

Supplementary Tables

Table S1. Docking score calculated by rigid-receptor docking before and after the MD simulations.

Binding Mode	Score before MD simulation	Score after MD simulation
1	-9.366	-12.479
2	-11.475	-12.170
3	-11.833	-12.351
4	-6.182	-11.430
5	-8.018	-11.272
6	-9.525	-11.708

Table S2. Occupancy of hydrogen bonds for Lp-PLA$_2$ substrates during the MD simulations

Substrate	HBond Donor	HBond Acceptor	Occupancy (%)
9:0 (COOH) PAF (PAF-azelaoyl)	His150 – side chain	Ligand – Oxygen atom (COOH)	49.42
	Tyr160 – side chain	Ligand – Oxygen atom (COOH)	79.88
	His272 – side chain	Ligand – Oxygen atom (COOH)	44.96
	Gln352 – side chain	Ligand – Oxygen atom (sn-1 either)	1.12
2:0 PAF	Gln352 – side chain	Ligand – Oxygen atom (sn-1 either)	10.44
4:0 PAF	Gln352 – side chain	Ligand – Oxygen atom (sn-1 either)	10.78
9:0 (COOH) PC azelaoyl-PC	His150 – side chain	Ligand – Oxygen atom (COOH)	12.50
	Tyr160 – side chain	Ligand – Oxygen atom (COOH)	80.54
	His272 – backbone	Ligand – Oxygen atom (COOH)	49.46
	Gln352 – side chain	Ligand – Oxygen atom (sn-1 carbonyl)	40.66
5:0 (COOH) PC succinoyl-PC	Tyr160 – side chain	Ligand – Oxygen atom (COOH)	99.90
	His272 – side chain	Ligand – Oxygen atom (COOH)	74.96
	Gln352 – side chain	Ligand – Oxygen atom (sn-1 carbonyl)	29.16
5:0 (ALDO) PC 5'-oxo-valeroyl-PC	Tyr160 – side chain	Ligand – Oxygen atom (ALDO)	71.06
	His272 – side chain	Ligand – Oxygen atom (ALDO)	42.50
	Gln352 – side chain	Ligand – Oxygen atom (sn-1 carbonyl)	66.42
9:0 (ALDO) PC 9'-oxo-nonanoyl-PC	His150 – side chain	Ligand – Oxygen atom (ALDO)	49.22
	Gln352 – side chain	Ligand – Oxygen atom (sn-1 carbonyl)	27.82

Table S3. Number and captions of movies

No	Caption
Movie 1	Optimization of the SB-402564 binding mode (BM1) in the Lp-PLA$_2$ active site.
Movie 2	Optimization of the SB-402564 binding mode (BM2) in the Lp-PLA$_2$ active site.
Movie 3	Optimization of the SB-402564 binding mode (BM3) in the Lp-PLA$_2$ active site.

Movie 4 Optimization of the SB-402564 binding mode (BM4) in the Lp-PLA_2 active site.

Movie 5 Optimization of the SB-402564 binding mode (BM5) in the Lp-PLA_2 active site.

Movie 6 Optimization of the SB-402564 binding mode (BM6) in the Lp-PLA_2 active site.

Movie 7 Alignment of all SB-402564 binding modes (BM1-6) in the Lp-PLA_2 active site.

Movie 8 MD simulation of Lp-PLA_2 in the presence of PAF (2:0) and membrane.

Movie 9 MD simulation of Lp-PLA_2 in the presence of PAF (2:0) in the active site.

Movie 10 MD simulation of Lp-PLA_2 in the presence of PAF (4:0) in the active site.

Movie 11 MD simulation of Lp-PLA_2 in the presence of PAF-azelaoyl (9:0, COOH) in the active site.

Movie 12 MD simulation of Lp-PLA_2 in the presence of azelaoyl-PC (9:0, COOH) in the active site.

Movie 13 MD simulation of Lp-PLA_2 in the presence of succinoyl-PC (5:0 (ALDO)) in the active site.

Movie 14 MD simulation of Lp-PLA_2 in the presence 5'-oxo-valeroyl-PC (5:0 (ALDO)) in the active site.

Movie 15 MD simulation of Lp-PLA_2 in the presence of 9'-oxo-nonanoyl-PC (9:0 (ALDO)) in the active site.

Movie 16 MD simulation of Lp-PLA_2 in the presence of F2-isoprostane-PC in the active site.

Movie 17 MD simulation that shows the flexible region 100-130 causing a conformational change that allows the enzyme to shift from a “closed” to an “open” conformation and vice versa depending on the environment.

References

1. Cao J, Hsu Y-H, Li S, Woods VL, & Dennis EA (2011) Lipoprotein-associated phospholipase A_2 interacts with phospholipid vesicles via a surface-disposed hydrophobic α-helix. *Biochemistry* 50:5314-5321.
2. Cao J, Hsu YH, Li S, Woods VL, Jr., & Dennis EA (2013) Structural basis of specific interactions of Lp-PLA_2 with HDL revealed by hydrogen deuterium exchange mass spectrometry. *J. Lipid Res.* 54:127-133.
3. Mouchlis VD, Chen Y, McCammon JA, & Dennis EA (2018) Membrane Allostery and Unique Hydrophobic Sites Promote Enzyme Substrate Specificity. *J. Am. Chem. Soc.* 140:3285-3291.
4. Mouchlis VD, Armando A, & Dennis EA (2019) Substrate-Specific Inhibition Constants for Phospholipase A_2 Acting on Unique Phospholipid Substrates in Mixed Micelles and Membranes Using Lipidomics. *J. Med. Chem.* 62:1999-2007.
5. Sastry GM, Adzhigirey M, Day T, Annabhimoju R, & Sherman W (2013) Protein and ligand preparation: parameters, protocols, and influence on virtual screening enrichments. *J. Comput.-Aided Mol. Des.* 27:221-234.
6. Halgren TA (2009) Identifying and characterizing binding sites and assessing druggability. *J. Chem. Inf. Model.* 49:377-389.
7. Halgren T (2007) New method for fast and accurate binding-site identification and analysis. *Chem. Biol. Drug. Des.* 69:146-148.
8. Samanta U & Bahnson BJ (2008) Crystal Structure of Human Plasma Platelet-activating Factor Acetylhydrolase: Structural implication to lipoprotein binding and catalysis. *J. Biol. Chem.* 283:31617-31624.
9. Halgren TA*, et al.* (2004) Glide: a new approach for rapid, accurate docking and scoring. 2. Enrichment factors in database screening. *J. Med. Chem.* 47:1750-1759.
10. Friesner RA*, et al.* (2004) Glide: a new approach for rapid, accurate docking and scoring. 1. Method and assessment of docking accuracy. *J. Med. Chem.* 47:1739-1749.
11. Sherman W, Day T, Jacobson MP, Friesner RA, & Farid R (2006) Novel procedure for modeling ligand/receptor induced fit effects. *J. Med. Chem.* 49:534-553.
12. Friesner RA*, et al.* (2006) Extra precision glide: docking and scoring incorporating a model of hydrophobic enclosure for protein-ligand complexes. *J. Med. Chem.* 49:6177-6196.
13. Lee J*, et al.* (2016) CHARMM-GUI Input Generator for NAMD, GROMACS, AMBER, OpenMM, and CHARMM/OpenMM Simulations Using the CHARMM36 Additive Force Field. *J Chem Theory Comput* 12:405-413.
14. Wu EL*, et al.* (2014) CHARMM-GUI Membrane Builder toward realistic biological membrane simulations. *J Comput Chem* 35:1997-2004.
15. Hidaka H*, et al.* (2007) Analysis of human serum lipoprotein lipid composition using MALDI-TOF mass spectrometry. *Ann. Clin. Lab. Sci.* 37:213-221.
16. Humphrey W, Dalke A, & Schulten K (1996) VMD: visual molecular dynamics. *J. Mol. Graph.* 14:33-38.
17. Phillips J*, et al.* (2005) Scalable molecular dynamics with NAMD. *J. Comput. Chem.* 26:1781-1802.

18. Adelman SA (1976) Generalized Langevin equation approach for atom/solid-surface scattering: General formulation for classical scattering off harmonic solids. *J. Chem. Phys.* 64:2375-2388.
19. Feller SE, Zhang Y, Pastor RW, & Brooks BR (1995) Constant pressure molecular dynamics simulation: the Langevin piston method. *J. Chem. Phys.* 103:4613-4621.
20. Jean-Paul R, Giovanni C, & Herman JCB (1977) Numerical integration of the cartesian equations of motion of a system with constraints: molecular dynamics of n-alkanes. *J. Comput. Phys.* 23:327-341.
21. Ulrich E*, et al.* (1995) A smooth particle mesh ewald method. *J. Chem. Phys.* 103:8577-8593.
22. Vanommeslaeghe K*, et al.* (2009) CHARMM general force field: A force field for drug-like molecules compatible with the CHARMM all-atom additive biological force fields. *J Comput. Chem.* 31:671-690.
23. Klauda JB*, et al.* (2010) Update of the CHARMM all-atom additive force field for lipids: validation on six lipid types. *J. Phys. Chem. B* 114:7830-7843.
24. Durrant JD, de Oliveira CA, & McCammon JA (2011) POVME: an algorithm for measuring binding-pocket volumes. *J. Mol. Graph. Model.* 29:773-776.
25. Liu QF*, et al.* (2016) Structural and Thermodynamic Characterization of Protein-Ligand Interactions Formed between Lipoprotein-Associated Phospholipase A_2 and Inhibitors. *J. Med. Chem.* 59:5115-5120.
